# The national burden of influenza‐like illness and severe respiratory illness overall and associated with nine respiratory viruses in South Africa, 2013–2015

**DOI:** 10.1111/irv.12949

**Published:** 2022-02-11

**Authors:** Stefano Tempia, Jocelyn Moyes, Adam L. Cohen, Sibongile Walaza, Meredith L. McMorrow, Florette K. Treurnicht, Orienka Hellferscee, Nicole Wolter, Anne von Gottberg, Halima Dawood, Ebrahim Variava, Cheryl Cohen

**Affiliations:** ^1^ Influenza Division Centers for Disease Control and Prevention Atlanta GA USA; ^2^ Influenza Program Centers for Disease Control and Prevention Pretoria South Africa; ^3^ Centre for Respiratory Diseases and Meningitis National Institute for Communicable Diseases of the National Health Laboratory Service Johannesburg South Africa; ^4^ MassGenics Duluth GA USA; ^5^ School of Public Health, Faculty of Health Sciences University of the Witwatersrand Johannesburg South Africa; ^6^ Global Immunization Monitoring and Surveillance Team, Expanded Programme on Immunization, Department of Immunization, Vaccines and Biological World Health Organization Geneva Switzerland; ^7^ Division of Virology, National Health Laboratory Service Charlotte Maxeke Johannesburg Academic Hospital Johannesburg South Africa; ^8^ School of Pathology, Faculty of Health Sciences University of the Witwatersrand Johannesburg South Africa; ^9^ Department of Medicine Pietermaritzburg Metropolitan Hospital Pietermaritzburg South Africa; ^10^ Department of Medicine University of KwaZulu‐Natal Pietermaritzburg South Africa; ^11^ Department of Medicine Klerksdorp‐Tshepong Hospital Complex Klerksdorp South Africa; ^12^ Department of Medicine, Faculty of Health Sciences University of the Witwatersrand Johannesburg South Africa; ^13^ Perinatal HIV Research Unit University of the Witwatersrand Johannesburg South Africa

**Keywords:** burden, respiratory viruses, South Africa

## Abstract

**Background:**

Estimates of the disease burden associated with different respiratory viruses are severely limited in low‐ and middle‐income countries, especially in Africa.

**Methods:**

We estimated age‐specific numbers and rates of medically and non‐medically attended influenza‐like illness (ILI) and severe respiratory illness (SRI) that were associated with influenza, respiratory syncytial virus (RSV), rhinovirus, human metapneumovirus, adenovirus, enterovirus and parainfluenza virus types 1–3 after adjusting for the attributable fraction (AF) of virus detection to illness in South Africa during 2013–2015. The base rates were estimated from five surveillance sites and extrapolated nationally.

**Results:**

The mean annual rates per 100,000 population were 51,383 and 4196 for ILI and SRI, respectively. Of these, 26% (for ILI) and 46% (for SRI) were medically attended. Among outpatients with ILI, rhinovirus had the highest AF‐adjusted rate (7221), followed by influenza (6443) and adenovirus (1364); whereas, among inpatients with SRI, rhinovirus had the highest AF‐adjusted rate (400), followed by RSV (247) and influenza (130). Rhinovirus (9424) and RSV (2026) had the highest AF‐adjusted rates among children aged <5 years with ILI or SRI, respectively, whereas rhinovirus (757) and influenza (306) had the highest AF‐adjusted rates among individuals aged ≥65 years with ILI or SRI, respectively.

**Conclusions:**

There was a substantial burden of ILI and SRI in South Africa during 2013–2015. Rhinovirus and influenza had a prominent disease burden among patients with ILI. RSV and influenza were the most prominent causes of SRI in children and the elderly, respectively.

## 1 INTRODUCTION

Despite the use of interventions such as antimicrobial drugs and vaccination against leading pneumonia‐causing pathogens, acute respiratory infections (ARIs) remain a major cause of death globally, especially among children aged <5 years.^1^ In addition, mild and severe‐non‐fatal episodes of ARI are responsible for a substantial burden on the healthcare systems and the society, through illness, absenteeism, and associated costs.

The Pneumonia Etiology Research for Child Health (PERCH) study conducted in seven low‐ and middle‐income countries, including South Africa, estimated that respiratory viruses were responsible for 61% of severe pneumonia cases (compared with 27% due to bacteria) among HIV‐uninfected children aged <5 years.^2^ This suggests that, as bacterial etiologies decline due to vaccination, respiratory viral causes may gain greater prominence.^2^ Nonetheless, the relative contribution of respiratory viruses in individuals of all ages remains poorly understood.

HIV infection is associated with increased severity of ARI and higher case‐fatality ratios, especially in older children and younger adults.^3^, ^4^ In South Africa, a country with an HIV prevalence of 12.7% in the general population and 23.5% in individuals aged 25–44 years in 2016,^5^ pneumonia and influenza were the leading causes of death among children aged <5 years and the 3rd and 5th causes of death among older individuals, resulting in 19,638 deaths annually in the same year.^6^ Whereas the etiology of hospitalized severe pneumonia among HIV‐uninfected children aged <5 years has been described in South Africa,^2^ the overall burden on the healthcare system and society of mild and severe respiratory illness overall and associated with respiratory viruses across age groups has not been quantified. Quantifying this burden may assist policy makers with allocation of resources and prioritization of interventions.

In this study, we sought to assess the mean annual national burden of medically and non‐medically attended influenza‐like illness (ILI) and severe respiratory illness (SRI) overall and associated with nine respiratory viruses in different age groups in South Africa during 2013–2015.

## 2 METHODS

### 2.1 Data sources

○ Data source 1: Respiratory viruses surveillance among patients with influenza‐like‐illness and SRI

We conducted active prospective hospital‐based surveillance among patients with SRI at three public hospitals in two provinces (Edendale Hospital in a peri‐urban area of KwaZulu‐Natal province and Klerksdorp and Tshepong Hospitals [the Klerksdorp‐Tshepong Hospital Complex, KTHC] in a peri‐urban area of North West province) during 2013–2015. A case of SRI was defined as a hospitalized person with symptoms of any duration who met age‐specific clinical inclusion criteria. A case in children aged 2 days to <3 months included any hospitalized patient with diagnosis of suspected sepsis or physician‐diagnosed acute lower respiratory tract infection irrespective of signs and symptoms. A case in children aged 3 months to <5 years included any hospitalized patient with physician‐diagnosed acute lower respiratory tract infection, including bronchitis, bronchiolitis, pneumonia, and pleural effusion. A case in individuals aged ≥5 years included any hospitalized patient presenting with manifestation of acute lower respiratory tract infection with fever (≥38°C) or history of fever and cough.

In addition, we conducted prospective surveillance for patients presenting with ILI at two outpatient clinics (Edendale Gateway Clinic, KwaZulu‐Natal province and Jouberton Clinic, North West province) located in the same catchment area as the abovementioned hospitals over the same study period. An ILI case was defined as an outpatient of any age presenting with either temperature ≥38°C or history of fever and cough of duration of ≤10 days.

We also enrolled persons presenting at the same outpatient clinics with no history of fever, respiratory, or gastrointestinal symptoms during the 14 days preceding the visit (hereafter referred to as controls). These individuals commonly presented to the clinics for visits such as dental procedures, family planning, well baby visits, voluntary HIV counseling and testing, or acute care for non‐febrile illnesses. We aimed to enroll one HIV‐infected and one HIV‐uninfected control every week in each clinic within each of the following age categories: <1, 1–4, 5–24, 25–44, 45–64, and ≥65 years.

The procedures of these surveillance programs have been previously described.^7^, ^8^, ^9^ In brief, study staff completed case report forms for all enrolled controls and ILI and SRI cases. Referral to hospital was recorded for all enrolled ILI cases. ILI cases that were referred to hospital were excluded from the analysis. Numbers of patients meeting the ILI and SRI case definitions and numbers enrolled were collected throughout the study period. Outpatient care prior to hospitalization was also recorded for enrolled SRI cases.

Respiratory specimens (i.e., nasopharyngeal aspirates for children aged <5 years and nasopharyngeal and oropharyngeal swabs from persons aged ≥5 years) were collected from all enrolled individuals (controls and ILI and SRI cases), placed in universal transport medium (Copan Diagnostics Inc., California, USA), stored at 4–8°C, and transported to the National Institute for Communicable Diseases (NICD) for testing within 72 h of collection. Specimens were tested for the presence of 10 respiratory viruses (influenza A and B viruses; parainfluenza virus [PIV] Types 1, 2, and 3; respiratory syncytial virus [RSV]; adenovirus; rhinovirus; human metapneumovirus [HMPV]; and enterovirus) using a multiplex real‐time reverse transcription polymerase chain reaction (PCR) assay.^10^ Influenza A‐positive samples were further subtyped.

HIV results were obtained from a combination of two sources: (i) patient clinical records when available and (ii) for consenting patients, a dried blood spot was tested at NICD. Testing included HIV enzyme‐linked immunosorbent assay (ELISA) for patients aged ≥18 months and PCR for children aged <18 months if the ELISA was reactive.

○ Data source 2: Healthcare utilization surveys (HUSs)

We obtained data on actual healthcare‐seeking behavior (including information on provider/institution where medical care was sought or not seeking medical care) among individuals with reported ILI and SRI from three HUSs conducted in South Africa.^11^, ^12^

○ Data source 3: Prevalence of risk factors for pneumonia and healthcare‐seeking behavior for ARI

We obtained the provincial‐level prevalence of known risk factors for pneumonia and the provincial data on healthcare‐seeking behavior among cases with ARI from the 2016 South Africa Demographic and Health Survey (DHS)^13^ and the THEMBISA model (specifically for the prevalence of HIV infection in the community).^14^

○ Data source 4: Population denominators

We obtained age‐ and year‐specific population denominators for the catchment area of the surveillance sites described in Data source 1 from projections of 2011 census data for South Africa.^15^ We also obtained the provincial age‐ and year‐specific population denominators from the same data source.

### 2.2 Estimation of the AF of respiratory viruses detection to illness

We used unconditional logistic regression to estimate the attributable fraction (AF) of respiratory viruses‐associated hospitalization and outpatient consultation by comparing the respiratory viruses detection rate among ILI or SRI cases with those of controls (outcome variables in the models). The AF was estimated from the virus specific odds ratio (OR) obtained from the multivariable regression models as follows:

(1) $$\mathrm{AF}=\frac{\text{OR}-1}{\text{OR}}*100.$$

Subsequently, we estimated the individual respiratory viruses detection rate associated with illness among ILI and SRI cases (*Infl*_*DetectRateIll*_) from the observed detection rate (*Infl*_*DetectRateObs*_) as follows:

(2) $${\text{Infl}}_{\text{DetectRateIll}}={\text{Infl}}_{\text{DetectRateObs}}*\mathrm{AF}.$$

This analysis was implemented overall and within the following age categories: <5, 5–44, and ≥45 years of age. All estimates were obtained from multivariable models that included the different respiratory viruses investigated in this study, HIV infection, and underlying medical conditions as covariates as previously described.^16^, ^17^

### 2.3 Estimation of the national disease burden associated with respiratory viruses

We reported mean annual estimates over the study period for any SRI and ILI as well as those associated with the respiratory viruses investigated in this study. Estimates were obtained overall and within the following age categories: <1, 1–4, 5–24, 25–44, 45–64, ≥65, <5, and ≥5 years of age. Rates were reported per 100,000 population. The details of the estimation approach are provided in Supporting Information and summarized below.

#### 2.3.1 Medically attended illness

To estimate the national number of respiratory viruses‐associated SRI hospitalizations, we used a four‐step approach. In Step 1, we estimated the SRI hospitalizations rates at the two hospitals mentioned above (Data source 1) during 2013–2015 as previously described,^18^ and we used the SRI hospitalization rates at the two sites as proxy for the corresponding provinces (considered to be the base provinces in our estimation approach). In Step 2, we estimated the SRI hospitalizations rates for the other seven provinces from the base provinces using a previously described methodology that leverages provincial differences in the prevalence of known risk factors for pneumonia and healthcare seeking behavior (Data source 3). In Step 3, we estimated the respiratory viruses‐associated SRI hospitalizations rates using available virological surveillance data (Data source 1) (i.e., detection rate of individual viruses adjusted for the estimated AF for each virus). In Step 4, we obtained the number of respiratory viruses‐associated SRI hospitalizations using the estimated respiratory viruses‐associated rates and the population at risk in each province (Data source 4).

To estimate the national number of respiratory viruses‐associated ILI outpatient consultations, we used an approach similar to that used for SRI cases, but we did not adjust for the provincial level risk factors for pneumonia.^18^

#### 2.3.2 Non‐medically attended illness

To estimate the national number and rates of respiratory viruses‐associated non‐medically attended ILI and SRI, we used the four‐step approach described above in conjunction with HUS data (Data source 2).^18^

### 2.4 Estimation of the confidence intervals

We obtained the 95% confidence intervals (CIs) for medically and non‐medically attended respiratory viruses‐associated illness using bootstrap resampling over 1000 replications for all parameters included in the calculations.^18^ This included (i) the age‐ and year‐specific ILI or SRI rates in the base provinces (Data source 1), (ii) the provincial prevalence of the risk factors for pneumonia from the DHS (Data source 3), (iii) the provincial proportion of ARI cases seeking care from the DHS (Data source 3), (iv) the age‐specific respiratory viruses detection rate among ILI or SRI cases tested (Data source 1), (v) the estimated AF for each virus, and (vi) the estimated proportion of individuals seeking care from the HUS (Data source 2). The lower and upper limits of the 95% CI were the 2.5^th^ and 97.5^th^ percentiles of the estimated values obtained from the 1000 resampled datasets, respectively.

## 3 RESULTS

### 3.1 Patients enrolled in surveillance

During January 2013–December 2015 we enrolled 10,602 individuals of which 10,149 (95.7%) had available data on age and underlying medical conditions as well as respiratory viruses and HIV results and were thus included for further analyses. Of these 10,602 individuals, 2119 (20.9%) were controls, 3672 (36.2%) were outpatients with ILI, and 4358 (42.9%) were inpatients with SRI. Children aged <5 years accounted for 38.0% (806/2119) of controls, 35.5% (1134/3672) of patients with ILI, and 37.5% (1632/4358) of patients with SRI. The HIV prevalence was 41.3% (874/2119) among controls (due to enrolment criteria), 25.1% (923/3672) among patients with ILI, and 50.1% (2183/4358) among patients with SRI.

### 3.2 Detection of respiratory viruses

**FIGURE 1:**
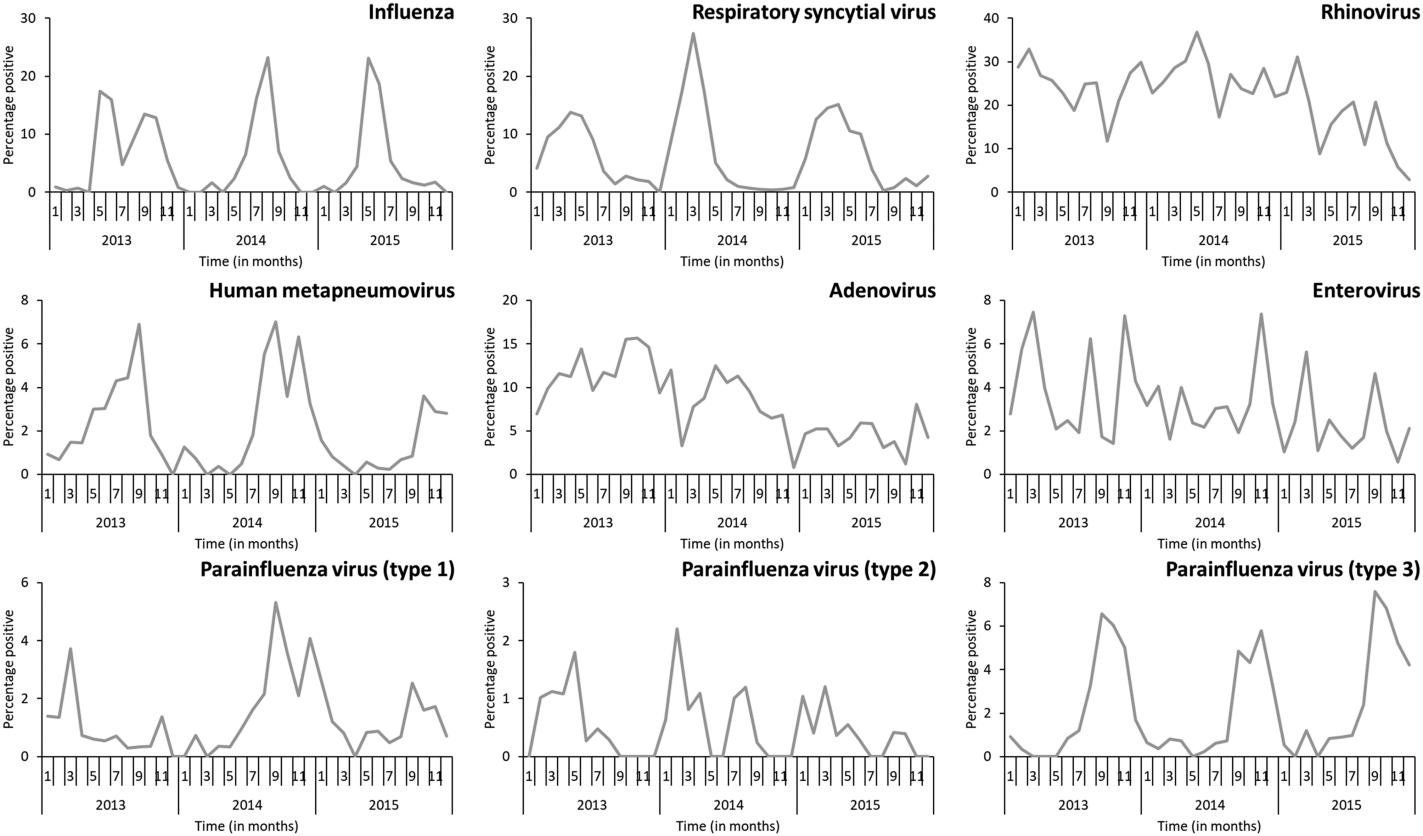
Circulation patterns of nine respiratory viruses, Klerksdorp and Pietermaritzburg, South Africa, 2013–2015

Among individuals of any age, the detection of the nine respiratory viruses investigated in this study ranged between 0.2% and 16.7% among controls, 0.6% and 26.2% among patients with ILI, and 0.6% and 22.1% among patients with SRI. Rhinovirus was the most detected virus among controls (16.7%; 354/2119) and patients with ILI (26.2%; 961/3672) or SRI (22.1%; 962/4358) (Tables 1, 2, and S1). Among controls, the most detected viruses following rhinovirus were adenovirus (7.1%; 150/2119) and enterovirus (2.4%; 51/2119). All other viruses were detected in <2% of samples in this group (Table S1). Following rhinovirus, the most detected viruses were influenza (13.1%; 480/3672) and adenovirus (8.9%; 327/3672) among patients with ILI (Tables 1 and S1) and RSV (9.9%; 432/4358) and adenovirus (8.8%; 385/4358) among patients with SRI (Table 2 and S1). The circulation patterns of the respiratory viruses investigated in this study are provided in Figure 1.

**TABLE 1:** Attributable fraction (AF), observed positivity proportion (OPP), and AF‐adjusted positivity proportion (AF‐adj. PP) of nine respiratory viruses among outpatients with influenza‐like illness, Klerksdorp and Pietermaritzburg, South Africa, 2013–2015

Age (in years)	Influenza			RSV		
	AF^a^ (95% CI)	OPP % (*n*/*N*)	AF‐adj. PP %	AF^a^ (95% CI)	OPP % (*n*/*N*)	AF‐adj. PP %
<1	91.0 (82.8–95.2)	7.3 (34/465)	6.6	78.9 (64.2–87.5)	9.9 (46/465)	7.8
1–4	91.0 (82.8–95.2)	14.9 (125/839)	13.6	78.9 (64.2–87.5)	8.9 (75/839)	7.0
5–24	92.8 (87.5–95.9)	17.2 (167/972)	16.0	65.0 (32.4–81.9)	2.7 (26/972)	1.8
25–44	92.8 (87.5–95.9)	13.1 (129/986)	12.2	65.0 (32.4–81.9)	3.1 (31/986)	2.0
45–64	71.4 (33.0–87.8)	6.5 (23/354)	4.6	74.2 (11.4–92.5)	2.5 (9/354)	1.9
≥65	71.4 (33.0–87.8)	3.6 (2/56)	2.6	74.2 (11.4–92.5)	3.6 (2/56)	2.7
All	90.8 (87.0–93.6)	13.1 (480/3672)	11.9	74.8 (63.4–82.7)	5.1 (189/3672)	3.8

Age (in years)	Rhinovirus			HMPV		
	AF^a^ (95% CI)	OPP % (*n*/*N*)	AF‐adj. PP %	AF^a^ (95% CI)	OPP % (*n*/*N*)	AF‐adj. PP %
<1	**42.9 (28.5–54.4)**	38.3 (178/465)	16.4	**79.9 (50.6–91.8)**	3.0 (14/465)	2.4
1–4	**42.9 (28.5–54.4)**	31.3 (263/839)	13.4	**79.9 (50.6–91.8)**	3.8 (32/839)	3.0
5–24	**61.0 (50.8–69.1)**	25.2 (245/972)	15.4	**76.3 (47.9–89.2)**	3.2 (31/972)	2.4
25–44	**61.0 (50.8–69.1)**	20.2 (199/986)	12.3	**76.3 (47.9–89.2)**	2.1 (21/986)	1.6
45–64	**71.7 (54.5–82.4)**	18.9 (67/354)	13.6	58.0 (−54.9 to 88.6)	2.0 (7/354)	1.2
≥65	**71.7 (54.5–82.4)**	16.1 (9/56)	11.5	58.0 (−54.9 to 88.6)	1.8 (1/56)	1.0
All	**53.1 (45.8–59.3)**	26.2 (961/3672)	13.9	**77.5 (61.7–86.7)**	2.9 (106/3672)	2.2

Age (in years)	Adenovirus			Enterovirus		
	AF^a^ (95% CI)	OPP % (*n*/*N*)	AF‐adj. PP %	AF^a^ (95% CI)	OPP % (*n*/*N*)	AF‐adj. PP %
<1	17.4 (−10.7 to 38.4)	9.5 (44/465)	1.7	**43.1 (12.5–63.0)**	6.9 (32/465)	3.0
1–4	17.4 (−10.7 to 38.4)	19.0 (159/839)	3.3	**43.1 (12.5–63.0)**	7.0 (59/839)	3.0
5–24	**49.4 (22.1–67.1)**	6.9 (67/972)	3.4	**73.7 (33.1–89.7)**	2.1 (20/972)	1.5
25–44	**49.4 (22.1–67.1)**	4.6 (45/986)	2.3	**73.7 (33.1–89.7)**	1.1 (11/986)	0.8
45–64	37.8 (−70.7 to 77.3)	3.1 (11/354)	1.2	91.5 (−77.0 to 99.6)	0.6 (2/354)	0.5
≥65	37.8 (−70.7 to 77.3)	1.8 (1/56)	0.7	91.5 (−77.0 to 99.6)	1.8 (1/56)	1.6
All	**20.7 (1.7–36.0)**	8.9 (327/3672)	1.8	**45.5 (23.1–61.4)**	3.4 (125/3672)	1.5

Age (in years)	PIV Types 1–3			PIV Type 1		
	AF^a^ (95% CI)	OPP % (*n*/*N*)	AF‐adj. PP %	AF^a^ (95% CI)	OPP % (*n*/*N*)	AF‐adj. PP %
<1	**67.2 (47.1–79.7)**	8.6 (40/465)	5.8	**66.6 (28.6–84.4)**	2.4 (11/465)	1.6
1–4	**67.2 (47.1–79.7)**	6.6 (55/839)	4.4	**66.6 (28.6–84.4)**	3.3 (28/839)	2.2
5–24	**70.6 (41.7–85.1)**	3.1 (30/972)	2.2	**71.1 (7.4–91.0)**	1.1 (11/972)	0.8
25–44	**70.6 (41.7–85.1)**	2.4 (24/986)	1.7	**71.1 (7.4–91.0)**	0.9 (9/986)	0.6
45–64	**80.7 (14.1–95.7)**	1.4 (5/354)	1.1	63.9 (−164.2 to 95.1)	0.6 (2/354)	0.4
≥65	**80.7 (14.1–95.7)**	5.4 (3/56)	4.4	63.9 (−164.2 to 95.1)	1.8 (1/56)	1.2
All	**67.3 (53.0–77.2)**	4.3 (157/3672)	2.9	**70.2 (45.6–83.6)**	1.7 (62/3672)	1.2

Age (in years)	PIV Type 2			PIV Type 3		
	AF^a^ (95% CI)	OPP % (*n*/*N*)	AF‐adj. PP %	AF^a^ (95% CI)	OPP % (*n*/*N*)	AF‐adj. PP %
<1	41.5 (−94.7 to 82.4)	1.1 (5/465)	0.5	**70.5 (42.3–84.9)**	5.2 (24/465)	3.7
1–4	41.5 (−94.7 to 82.4)	0.7 (6/839)	0.3	**70.5 (42.3–84.9)**	2.5 (21/839)	1.8
5–24	67.2 (−42.8 to 92.5)	0.7 (7/972)	0.5	**67.0 (1.05–87.2)**	1.2 (12/972)	0.8
25–44	67.2 (−42.8 to 92.5)	0.2 (2/986)	0.1	**67.0 (15.0–87.2)**	1.4 (14/986)	0.9
45–64	91.6 (−75.8 to 99.6)	0.3 (1/354)	0.3	65.1 (−196.1 to 95.9)	0.6 (2/354)	0.4
≥65	91.6 (−75.8 to 99.6)	3.6 (2/56)	3.3	65.1 (−196.1 to 95.9)	1.8 (1/56)	1.2
All	**60.5 (2.7–84.0)**	0.6 (23/3672)	0.4	**65.4 (42.8–79.1)**	2.0 (74/3672)	1.3

Abbreviations: HMPV, human metapneumovirus; PIV, parainfluenza virus; RSV, respiratory syncytial virus.

^a^ Attributable fraction calculated for individuals aged <5, 5–44, and ≥45 years of age and used to adjust the observed positivity proportion among individuals aged <1 and 1–4; 5–24 and 25–44; and 45–64 and ≥65 years, respectively.

**TABLE 2:** Attributable fraction (AF), observed positivity proportion (OPP), and AF‐adjusted positivity proportion (AF‐adjusted PP) of nine respiratory viruses among inpatients with severe respiratory illness, Klerksdorp and Pietermaritzburg, South Africa, 2013–2015

Age (in years)	Influenza			RSV		
	AF^a^ (95% CI)	OPP % (*n*/*N*)	AF‐adj. PP %	AF^a^ (95% CI)	OPP % (*n*/*N*)	AF‐adj. PP %
<1	**79.4 (62.7–88.7)**	4.0 (41/1018)	3.2	**92.8 (88.5–95.5)**	29.4 (299/1018)	27.3
1–4	**79.4 (62.7–88.7)**	7.3 (45/614)	5.8	**92.8 (88.5–95.5)**	13.0 (80/614)	12.1
5–24	**71.8 (44.0–85.7)**	4.2 (14/330)	3.0	43.1 (−22.3 to 73.5)	3.0 (10/330)	1.3
25–44	**71.8 (44.0–85.7)**	4.1 (55/1345)	2.9	43.1 (−22.3 to 73.5)	1.8 (24/1345)	0.8
45–64	**59.4 (10.2–81.6)**	4.1 (34/824)	2.4	54.5 (−45.7 to 85.8)	1.5 (12/824)	0.8
≥65	**59.4 (10.2–81.6)**	4.8 (11/227)	2.9	54.5 (−45.7 to 85.8)	3.1 (7/227)	1.7
All	**73.3 (61.1–81.7)**	4.6 (200/4358)	3.4	**91.9 (88.4–94.4)**	9.9 (432/4358)	9.1

Age (in years)	Rhinovirus			HMPV		
	AF^a^ (95% CI)	OPP % (*n*/*N*)	AF‐adj. PP %	AF^a^ (95% CI)	OPP % (*n*/*N*)	AF‐adj. PP %
<1	**50.5 (38.7–60.0)**	31.4 (320/1018)	15.9	**87.7 (71.7–94.6)**	4.7 (48/1018)	4.1
1–4	**50.5 (38.7–60.0)**	38.6 (237/614)	19.5	**87.7 (71.7–94.6)**	4.2 (26/614)	3.7
5–24	**55.2 (40.9–66.0)**	27.6 (91/330)	15.2	17.3 (−160.5 to 73.8)	0.6 (2/330)	0.1
25–44	**55.2 (40.9–66.0)**	15.5 (208/1345)	8.6	17.3 (−160.5 to 73.8)	0.7 (10/1345)	0.1
45–64	**36.9 (1.6–59.5)**	10.6 (87/824)	3.9	24.2 (−158.0 to 77.7)	0.7 (6/824)	0.2
≥65	**36.9 (1.6–59.5)**	8.4 (19/227)	3.1	24.2 (−158.0 to 77.7)	2.2 (5/227)	0.5
All	**47.4 (39.0–54.6)**	22.1 (962/4358)	10.5	**78.1 (62.3–87.2)**	2.2 (97/4358)	1.7

Age (in years)	Adenovirus			Enterovirus		
	AF^a^ (95% CI)	OPP % (*n*/*N*)	AF‐adj. PP %	AF^a^ (95% CI)	OPP % (*n*/*N*)	AF‐adj. PP %
<1	**39.0 (20.5–53.2)**	12.5 (127/1018)	4.9	**45.9 (19.5–63.6)**	5.6 (57/1018)	2.6
1–4	**39.0 (20.5–53.2)**	26.9 (165/614)	10.5	**45.9 (19.5–63.6)**	9.0 (55/614)	4.1
5–24	29.5 (−19.0 to 58.2)	3.6 (12/330)	1.1	**80.0 (40.4–93.3)**	2.1 (7/330)	1.7
25–44	29.5 (−19.0 to 58.2)	4.0 (54/1345)	1.2	**80.0 (40.4–93.3)**	0.7 (10/1345)	0.6
45–64	29.1 (−71.8 to 70.7)	2.4 (20/824)	0.7	82.9 (−225.6 to 99.1)	0.2 (2/824)	0.2
≥65	29.1 (−71.8 to 70.7)	3.1 (7/227)	0.9	82.9 (−225.6 to 99.1)	0.9 (2/227)	0.7
All	**26.4 (9.0–40.5)**	8.8 (385/4358)	2.3	**46.9 (24.7–62.6)**	3.1 (133/4358)	1.5

Age (in years)	PIV Types 1–3			PIV Type 1		
	AF^a^ (95% CI)	OPP % (*n*/*N*)	AF‐adj. PP %	AF^a^ (95% CI)	OPP % (*n*/*N*)	AF‐adj. PP %
<1	**66.3 (47.7–78.3)**	7.7 (78/1018)	5.1	**55.8 (6.0–79.2)**	1.9 (19/1018)	1.1
1–4	**66.3 (47.7–78.3)**	8.3 (51/614)	5.5	**55.8 (6.0–79.2)**	2.8 (17/614)	1.6
5–24	50.7 (−6.5 to 77.2)	3.0 (10/330)	1.5	64.0 (−27.3 to 89.8)	1.2 (4/330)	0.8
25–44	50.7 (−6.5 to 77.2)	2.2 (30/1345)	1.1	64.0 (−27.3 to 89.8)	0.9 (12/1345)	0.6
45–64	64.5 (−36.2 to 90.7)	1.5 (12/824)	1.0	17.5 (−447.8 to 87.6)	0.4 (3/824)	0.1
≥65	64.5 (−36.2 to 90.7)	2.6 (6/227)	1.7	17.5 (−447.8 to 87.6)	0.9 (2/227)	0.2
All	**68.7 (55.1–78.2)**	4.3 (187/4358)	3.0	**62.7 (31.0–79.9)**	1.3 (57/4358)	0.8

Age (in years)	PIV Type 2			PIV Type 3		
	AF^a^ (95% CI)	OPP % (*n*/*N*)	AF‐adj. PP %	AF^a^ (95% CI)	OPP % (*n*/*N*)	AF‐adj. PP %
<1	36.0 (−112.9 to 80.7)	0.3 (3/1018)	0.1	**72.9 (51.5–84.9)**	5.8 (59/1018)	4.2
1–4	36.0 (−112.9 to 80.7)	1.1 (7/614)	0.4	**72.9 (51.5–84.9)**	4.4 (27/614)	3.2
5–24	34.9 (−194.4 to 85.6)	0.9 (3/330)	0.3	31.8 (−124.3 to 79.3)	0.9 (3/330)	0.3
25–44	34.9 (−194.4 to 85.6)	0.5 (7/1345)	0.2	31.8 (−124.3 to 79.3)	0.8 (11/1345)	0.3
45–64	75.5 (−352.0 to 98.7)	0.5 (4/824)	0.4	58.1 (−144.8 to 92.8)	0.6 (5/824)	0.3
≥65	75.5 (−352.0 to 98.7)	0.4 (1/227)	0.3	58.1 (−144.8 to 92.8)	1.3 (3/227)	0.8
All	53.0 (−14.5 to 80.7)	0.6 (25/4358)	0.3	**73.5 (56.6–83.8)**	2.5 (108/4358)	1.8

Abbreviations: HMPV, human metapneumovirus; PIV, parainfluenza virus; RSV, respiratory syncytial virus.

^a^ Attributable fraction calculated for individuals aged <5, 5–44, and ≥45 years of age and used to adjust the observed positivity proportion among individuals aged <1 and 1–4; 5–24 and 25–44; and 45–64 and ≥65 years, respectively.

### 3.3 Respiratory viruses AF and AF‐adjusted prevalence

Among individuals of any age, the AF of the nine respiratory viruses investigated in this study ranged between 20.7% and 90.8% among patients with ILI (Table 1) and 26.4% and 91.9% among patients with SRI (Table 2). Influenza (90.8%), HMPV (77.5%), and RSV (74.8%) had the highest AF among patients with ILI (Table 1), and RSV (91.9%), HMPV (78.1%), and PIV type 3 (73.5%) had the highest AF among patients with SRI (Table 2).

**FIGURE 2:**
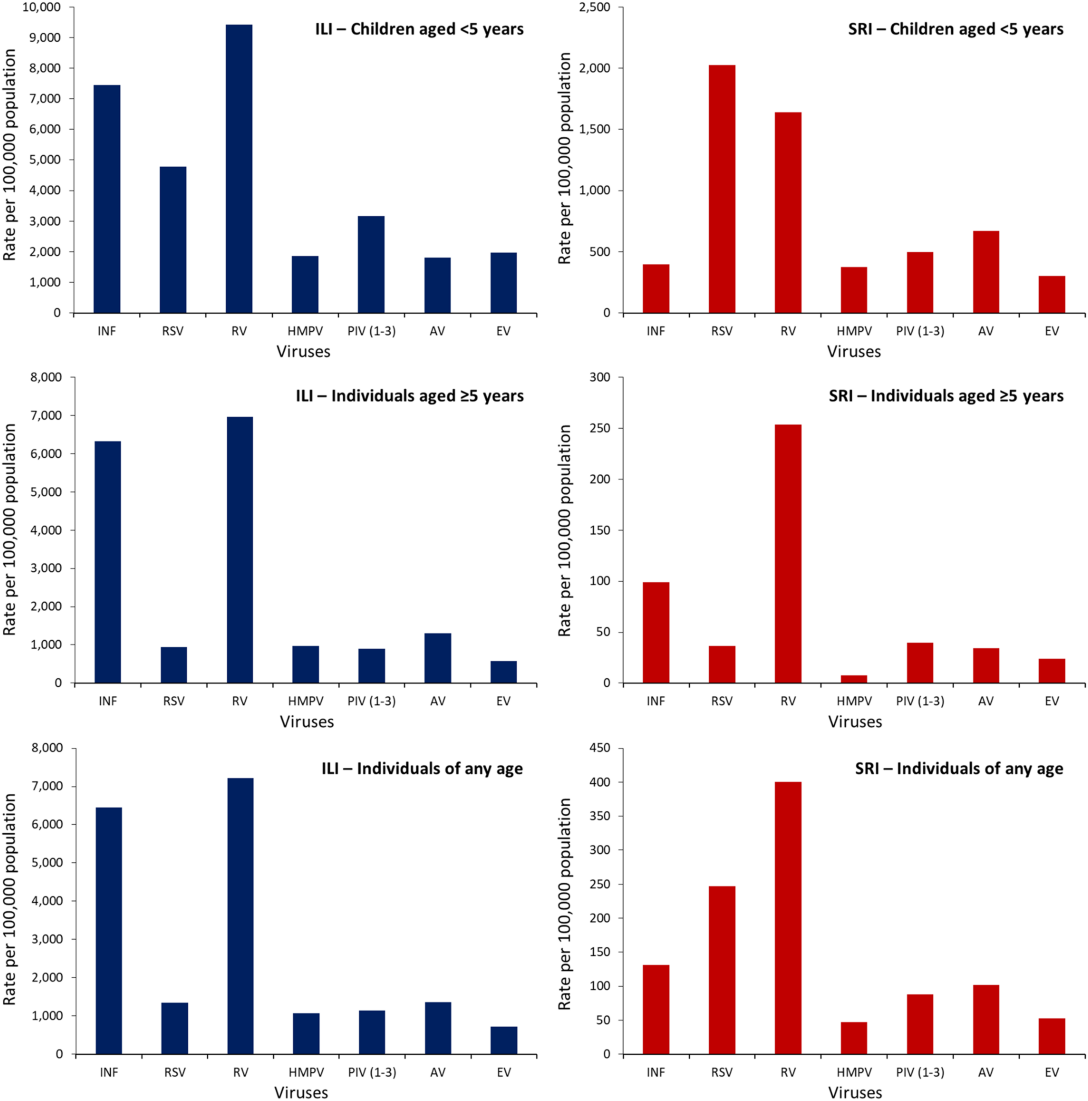
National estimates of mean annual rates of medically and non‐medically attended influenza‐like illness (ILI) and severe respiratory illness (SRI) associated with seven respiratory viruses in South Africa, 2013–2015 (INF: influenza virus; RSV: respiratory syncytial virus, RV: rhinovirus; HMPV: human metapneumovirus, PIV (1‐3): parainfluenza virus types 1‐3; AV: adenovirus; EV: enterovirus)

**FIGURE 3:**
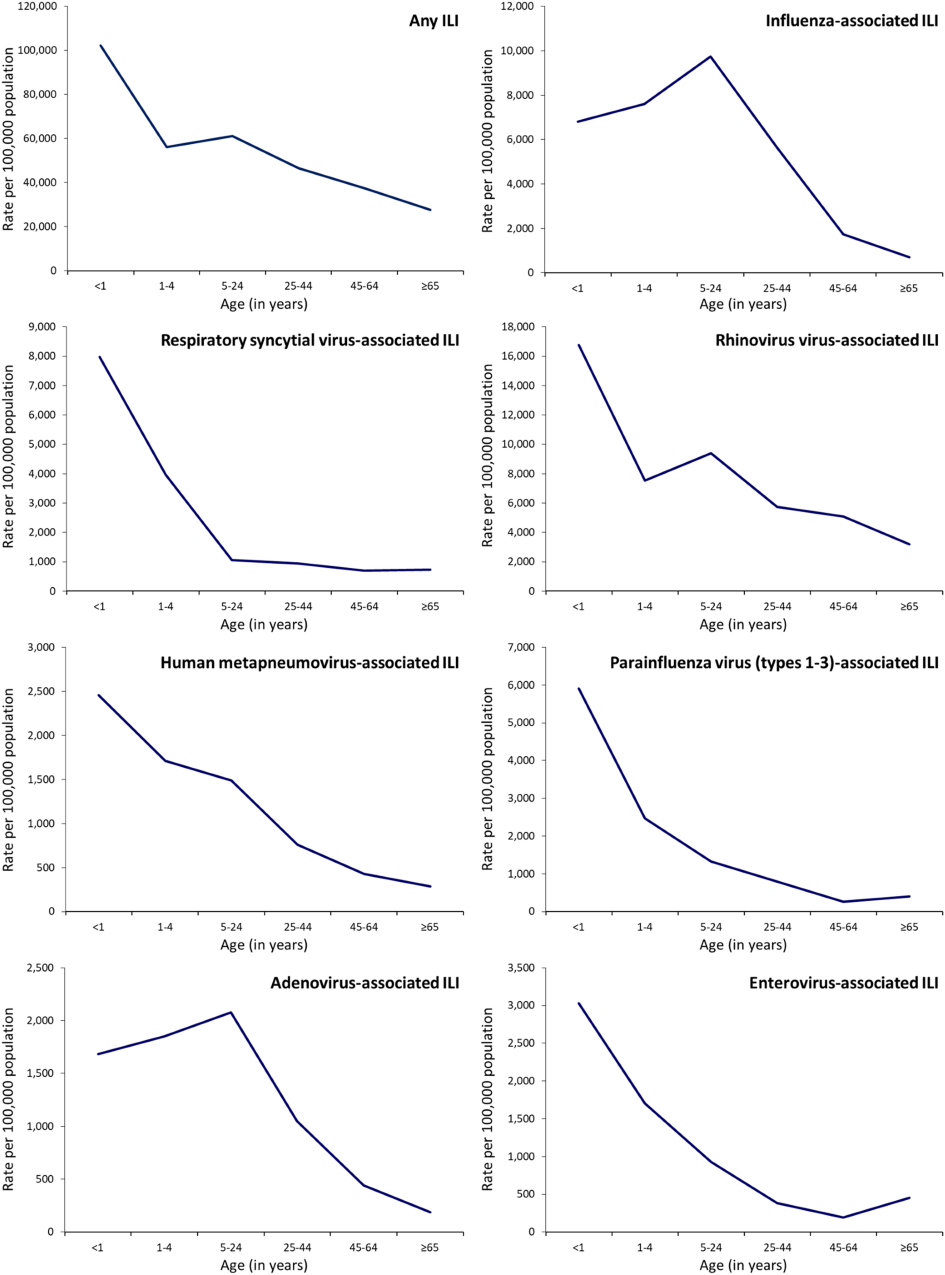
National estimates of mean annual rate of any medically and non‐medically attended influenza‐like illness (ILI) and those associated with seven respiratory viruses by age groups in South Africa, 2013–2015

**FIGURE 4:**
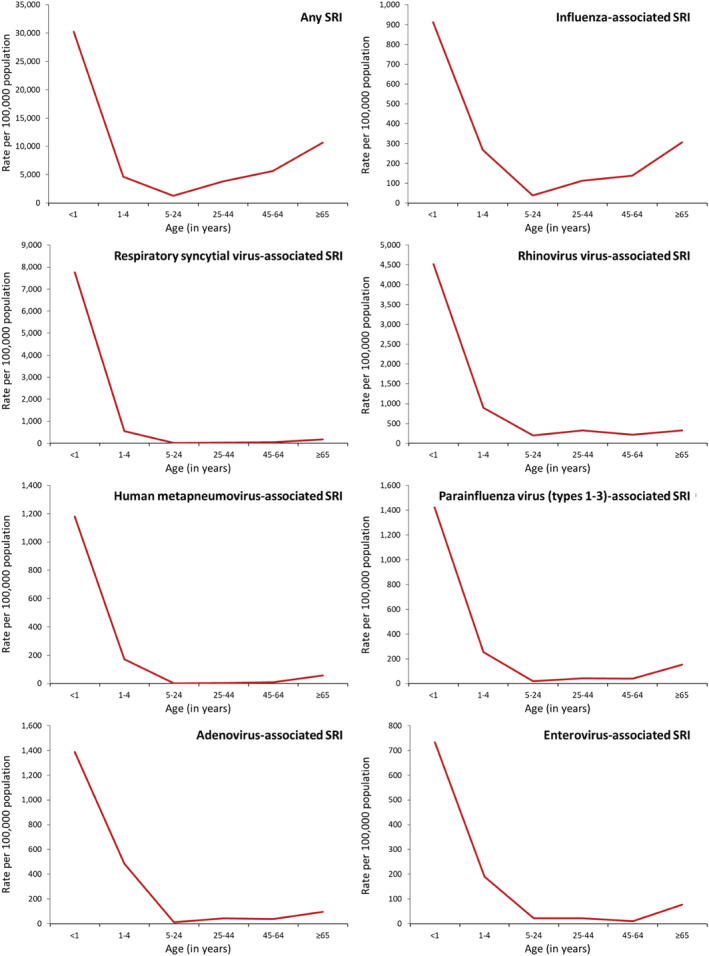
National estimates of mean annual rate of any medically and non‐medically attended severe respiratory illness (SRI) and those associated with seven respiratory viruses by age groups in South Africa, 2013–2015

**FIGURE 5:**
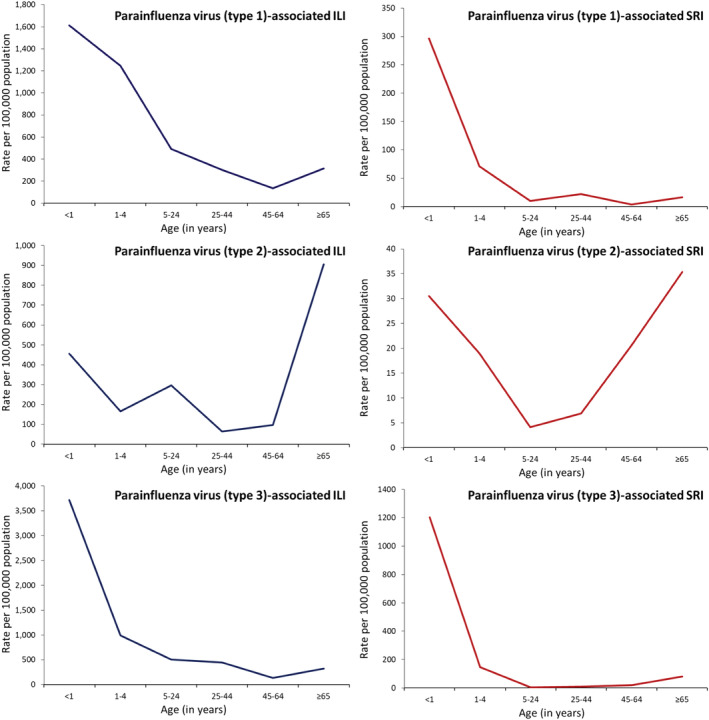
National estimates of mean annual rates of medically and non‐medically attended influenza‐like illness (ILI) and severe respiratory illness (SRI) associated with parainfluenza virus types 1–3 by age groups in South Africa, 2013–2015

Among patients of any age with ILI, rhinovirus (13.9%) had the highest AF‐adjusted prevalence followed by influenza (11.9%) and RSV (3.8%) (Table 1). Among patients of any age with SRI, rhinovirus (10.5%) also had the highest AF‐adjusted prevalence followed by RSV (9.1%) and influenza (3.4%) (Table 2). The AF‐adjusted prevalence was used to estimate the virus‐specific rates, which are provided in Figures 2, 3, 4, 5.

### 3.4 Burden of ILI and that associated with respiratory viruses

During 2013–2015, among individuals of any age, the estimated mean annual number of ILI episodes was 27,773,390 (rate: 51,383 per 100,000 population) (Table S2) of which 7,232,910 (26.0%; rate: 13,381 per 100,000 population) were medically attended (Table S3). The ILI rates were highest in infants and declined with increasing age (Figure 3 and Table S2). Overall, among patients with ILI, rhinovirus had the highest rate (7221 per 100,000 population) followed by influenza (6443 per 100,000 population) and adenovirus (1364 per 100,000 population) (Figure 2 and Table S2). A similar ranking was observed among individuals aged ≥5 years (Figure 2 and Table S2). Among children aged <5 years, rhinovirus had the highest rate (9424 per 100,000 population) followed by influenza (7440 per 100,000 population) and RSV (4774 per 100,000 population). The respiratory viruses‐associated ILI rates were highest in infants and declined with increasing age with the exception of influenza and adenovirus where the highest rates were among individuals aged 5–24 years (Figure 3 and Table S2) and PIV Type 2 where the highest rate was among individuals aged ≥65 years (Figure 5 and Table S8). The rates of medically and non‐medically attended respiratory viruses‐associated ILI are provided in Tables S3, S4, and S8.

### 3.5 Burden of SRI and that associated with respiratory viruses

During 2013–2015, among individuals of any age, the estimated mean annual number of SRI episodes was 2,268,125 (rate: 4196 per 100,000 population) (Table S5) of which 1,051,701 (46.4%; rate: 1945 per 100,000 population) were medically attended (Table S6). The SRI rates were highest in infants aged <1 year followed by individuals aged ≥65 years (Figure 4 and Table S5). Overall, among patients with SRI, rhinovirus had the highest rate (400 per 100,000 population) followed by RSV (247 per 100,000 population) and influenza (130 per 100,000 population) (Figure 2 and Table S5). Among children aged <5 years, RSV had the highest rate (2026 per 100,000 population) followed by rhinovirus (1638 per 100,000 population) and adenovirus (667 per 100,000 population) (Figure 2 and Table S5). Among individuals aged ≥5 years, rhinovirus had the highest rate (253 per 100,000 population) followed by influenza (98 per 100,000 population) and PIV Types 1–3 (39 per 100,000 population) (Figure 2 and Table S5). The respiratory viruses‐associated SRI rates were highest in infants aged <1 year followed by children aged 1–4 years with the exception of influenza where the second highest rate was among individuals aged ≥65 years (Figure 4 and Table S5) and PIV Type 2 where the highest rate was among individuals aged ≥65 years followed by infants aged <1 year (Figure 5 and Table S8). The rates of medically and non‐medically attended respiratory viruses‐associated SRI are provided in Tables S6, S7, and S9.

## 4 DISCUSSION

We estimated the AF of nine respiratory viruses among patients with ILI and SRI as well as the mean annual number and rates of these syndromes and that associated with respiratory viruses after adjusting for the estimated AF in South Africa during 2013–2015. The burden of ILI (mean annual rate: 51,383 per 100,000 population) and SRI (mean annual rate: 4196 per 100,000 population) was substantial. Twenty six percent of ILI and 46% of SRI episodes were medically attended, indicating a heavy burden of these syndromes on the healthcare system.

Estimates of the burden of ILI in Africa are scarce. Our estimated rates of medically attended ILI (13,381 per 100,000 population) are similar to those reported in a study conducted in the Democratic Republic of Congo (DRC) (11,890 per 100,000 population).^19^ Our estimated rates of medically attended SRI (1945 per 100,000 population) are higher than those estimated in other countries for severe acute respiratory illness (SARI): DRC (795 per 100,000 population),^19^ Kenya (350 per 100,000 population),^20^ Rwanda (571 per 100,000 population),^21^ Uganda (267–647 per 100,000 population),^22^ and Zambia (843 per 100,000 population).^23^ It should be noted that the SARI case definition used in these studies included patients with a symptom duration of 7 (mostly) or 10 days, whereas our SRI case definition included patients with symptoms of any duration, which would include more patients. In a study conducted in South Africa, the rates of influenza‐associated SRI (67 per 100,000 population) were 2.6 times higher than those of influenza associated SARI with symptoms duration of 7 days.^24^ Besides the difference in case definitions, difference in rates in different settings can also be attributed to differences in access to healthcare, healthcare‐seeking behavior, and the prevalence of risk factors for pneumonia in the general population such as HIV and other underlying medical conditions.

In our study, among patients with ILI, the most commonly detected virus was rhinovirus, followed by influenza and adenovirus. These viruses were detected at high frequencies also in other studies conducted in Africa among outpatients with respiratory illness.^25^, ^26^, ^27^, ^28^, ^29^ Rhinovirus, RSV, and adenovirus were the most commonly detected viruses among patients with SRI in our study, and, although variability was observed in different settings, these viruses were frequently detected among hospitalized patients with SARI/ARI in other studies from African countries.^30^, ^31^, ^32^, ^33^

Among patient with ILI the AF of influenza, RSV, HMPV, and PIV Types 1–3 were generally high (range: 68.7–91.9%), whereas those of adenovirus and enterovirus were generally low (range: 16.4–46.9%). A similar pattern was observed among patients with SRI, and this has been reported in other studies.^10^, ^16^, ^17^

Rhinovirus was the most detected virus among patients with ILI and SRI, but its AF was moderate (<55% for both syndromes among individuals of any age). The AF of rhinovirus found in this study was similar to those reported in other studies (range: 39.8–59.2%).^10^, ^16^ Despite its moderate AF, the AF‐adjusted prevalence of rhinovirus remained the highest (compared with those of other viruses) among patients of any age with ILI or SRI, due to the high level of detection. This was reported also in another study conducted in South Africa.^10^ Rhinovirus has been well described as one of the most common causes of common cold^34^; however, its role as causal agent of SRI is less well described. In our study, only RSV had an AF‐adjusted prevalence higher than that of rhinovirus among children aged <5 years with SRI. In the PERCH study, RSV had the highest AF and associated etiological fraction among HIV‐uninfected children aged <5 years with severe pneumonia.^2^ In the same study, rhinovirus had varying detection frequency and etiological fraction in the different participating countries.^2^ Nonetheless, in the pooled analysis, rhinovirus had a moderate but significant AF and the second highest etiological fraction (after RSV) among the investigated viruses, which is similar to the findings of our study. Studies on the etiological fraction of rhinovirus among patients aged >5 years with SRI are scarce. In our study, the AF‐adjusted prevalence of rhinovirus among patients aged >5 years with SRI was the highest, and it was similar to that observed in other South African studies.^10^, ^16^ Studies in this group of patients in other settings are warranted.

Among patients with ILI, the rates associated with the different viruses evaluated in this study were highest among children aged <1 and 1–4 years, with the exception of influenza and adenovirus, where the highest rates were among individuals aged 5–24 years and PIV Type 2 where the highest rates were among individuals aged ≥65 years. Young children have been described to be particularly susceptible to viral respiratory infections,^3^ and a prominent role of school‐aged children and young adults in the transmission of influenza virus has been described in other studies.^35^ Among patients with SRI, the rates associated with the different viruses evaluated in this study were also the highest among young children, with the exception of PIV Type 2 where the highest rates were among individuals aged ≥65 years. When compared with other viruses, RSV had the highest rates among children aged <5 years, whereas influenza had the highest rates among individuals aged ≥65 years. A heavy burden of these two pathogens in these age groups has been described in other studies.^8^, ^18^

This study has limitations that warrant discussion. First, the base incidence estimates were obtained from five surveillance sites and extrapolated nationally. Whereas the extrapolation approach used in this study has been used in several other similar studies,^18^, ^19^, ^20^, ^21^, ^22^, ^23^ the potential geographic differences may not be fully accounted for in the extrapolation approach. Second, the WHO ILI case definition used in this study does not capture the entire clinical spectrum of mild respiratory illness. This has been well described for influenza where only 30–50% of patients with mild influenza illness present with fever and cough.^35^, ^36^, ^37^ This proportion is poorly understood for other respiratory viruses, but an underestimate of the burden of mild illness associated with the different respiratory viruses is likely to occur when restricting the identification of cases among patients with ILI. The SRI case definition used in this study is broader than the SARI case definition recommended by WHO for influenza surveillance, but some severe respiratory cases may also have been missed using our SRI case definition. Hence, our estimates should be considered minimum estimates of the total burden of these respiratory viruses. Third, we did not test for the full spectrum of respiratory viruses nor for bacteria, and we collected only upper respiratory tract specimens. This hindered our ability to account for the full spectrum of respiratory pathogens and the results from multiple specimens (as implemented in other studies such as PERCH) in our analysis. Lastly, studies such as ours aim to provide a more “accurate” estimate of the disease burden associated with different pathogens by estimating the fraction of pathogens detection that is not associated with illness using controls. When the etiological fraction of different pathogens to a syndrome is estimated, one pathogen is then “assigned as the cause” of the observed illness. Nonetheless, there is a potential inherent “bias” in such approach, in that the observed illness may be the result of the interaction of multiple pathogens.^2^ Such limitation should be acknowledged when interpreting the results of studies like ours and the estimated burden associated to a specific pathogen should not be considered completely mutually exclusive from that of others. Despite this limitation, these studies have value to contextualize the detection of different pathogens as causal of an observed illness and to provide an indication of the relative contribution of different pathogens to a syndrome, whether interaction of different pathogens occurs or not.

In conclusion, there was a substantial burden of ILI and SRI in South Africa before the COVID‐19 pandemic. Our study suggests that, among the investigated viruses, influenza and rhinovirus had a prominent disease burden among patients with ILI. The results of our study support the results of other studies that identified RSV as the most prominent cause of SRI in children,^2^ whereas influenza has a substantial role in SRI in the elderly. The high burden of disease caused by these pathogens lends support to initiatives to accelerate the development of specific preventive interventions such as vaccination and expanded use of vaccination where vaccines exist. Ongoing surveillance is needed in the context of the COVID‐19 pandemic as mitigation measures have impacted the circulation of respiratory viruses in South Africa and in other countries globally.^38^, ^39^

## AUTHOR CONTRIBUTIONS

**Stefano Tempia:** Conceptualization; data curation; formal analysis; investigation; methodology; validation; visualization. **Jocelyn Moyes:** Conceptualization; investigation; methodology. **Adam Cohen:** Conceptualization; investigation; methodology. **Sibongile Walaza:** Conceptualization; data curation; investigation; methodology. **Meredith McMorrow:** Conceptualization; investigation; methodology. **Florette Treurnicht:** Conceptualization; investigation; methodology. **Orienka Hellferscee:** Conceptualization; investigation; methodology. **Nicole Wolter:** Conceptualization; investigation; methodology. **Anne von Gottberg:** Conceptualization; investigation; methodology. **Halima Dawood:** Conceptualization; investigation; methodology. **Ebrahim Variava:** Conceptualization; investigation; methodology. **Cheryl Cohen:** Conceptualization; investigation; methodology.

## DISCLAIMER

The findings and conclusions in this report are those of the authors and do not necessarily represent the official position of the US Centers for Disease Control and Prevention, USA or the National Institute for Communicable Diseases, South Africa.

### PEER REVIEW

The peer review history for this article is available at https://publons.com/publon/10.1111/irv.12949.

## Supporting information


**Data S1:** Supporting Information

## Data Availability

The data that support the findings of this study are available from the corresponding author upon reasonable request.
