# Supplementary material for: The national burden of influenza‐like illness and severe respiratory illness overall and associated with nine respiratory viruses in South Africa, 2013–2015
Source: Influenza Other Respir Viruses. 2022 Feb 11;16(3):438–51. doi: 10.1111/irv.12949 (PMC8983907; doi:10.1111/irv.12949)
Supplement: Supplementary file 1 — Data S1: Supporting Information [file IRV-16-438-s001.docx]

**The National Burden of Influenza-Like Illness and Severe Respiratory Illness Overall and Associated with Nine Respiratory Viruses in South Africa, 2013-2015 (Supplementary Material)**

**Authors**

Stefano Tempia^1,2,3,4,5^, Jocelyn Moyes^3,5^, Adam L. Cohen^1,6^, Sibongile Walaza^3,5^, Meredith L. McMorrow^1,2^, Florette K. Treurnicht^3,7^, Orienka Hellferscee^3,8^, Nicole Wolter^3,8^, Anne von Gottberg^3,8^, Halima Dawood^9,10^, Ebrahim Variava^11,12,13^, Cheryl Cohen^3,5^

**Affiliations**

^1^Influenza Division, Centers for Disease Control and Prevention, Atlanta, Georgia, United States of America.

^2^Influenza Program, Centers for Disease Control and Prevention, Pretoria, South Africa.

^3^Centre for Respiratory Diseases and Meningitis, National Institute for Communicable Diseases of the National Health Laboratory Service, Johannesburg, South Africa.

^4^MassGenics, Duluth, Georgia, Unites States of America.

^5^School of Public Health, Faculty of Health Sciences, University of the Witwatersrand, Johannesburg, South Africa.

^6^Global Immunization Monitoring and Surveillance Team, Expanded Programme on Immunization, Department of Immunization, Vaccines and Biological, World Health Organization, Geneva, Switzerland.

^7^Division of Virology, National Health Laboratory Service, Charlotte Maxeke Johannesburg Academic Hospital, Johannesburg, South Africa

^8^School of Pathology, Faculty of Health Sciences, University of the Witwatersrand, Johannesburg, South Africa.

^9^Department of Medicine, Pietermaritzburg Metropolitan Hospital, Pietermaritzburg, South Africa.

^10^Department of Medicine, University of KwaZulu-Natal, Pietermaritzburg, South Africa.

^11^Department of Medicine, Klerksdorp-Tshepong Hospital Complex, Klerksdorp, South Africa.

^12^Department of Medicine, Faculty of Health Sciences, University of the Witwatersrand, Johannesburg, South Africa.

^13^Perinatal HIV Research Unit, University of the Witwatersrand, Johannesburg, South Africa.

**METHODS**

**Estimation of the national disease burden associated with respiratory viruses**

***Medically attended illness***

To estimate the national number and rates of medically attended respiratory viruses-associated ILI and SRI we used a four-step approach described below [[[1]](#endnote-1)].

- *Step 1.a: Estimation of ILI and SRI rates in the base provinces (North West and KwaZulu-Natal)*

To estimate the SRI hospitalizations rates for the Edendale and KTHC hospitals we used a previously described methodology for these two sentinel sites [1]. The equation used to estimate the SRI hospitalization rates is provided below [1].

$SRI_{Rate_{i}}=\frac{SRI_{Enrolled_{i}}*\frac{7}{5}*\frac{1}{X_{SRI_{i}}}*\frac{1}{HUS_{SRI}}}{Pop_{i}}$ (1)

Where $SRI_{Rate_{i}}$ is the estimated age-specific SRI hospitalization rate in age group *i*; $SRI_{Enrolled_{i}}$is the age-specific number of SRI cases enrolled (data source 1); 7/5 is the coefficient used to adjust for non-enrolment over weekends; $X_{SRI_{i}}$ is the age-specific proportion of all eligible SRI cases that were enrolled (obtained from study logs - data source 1); *HUS_SRI_* is the proportion of SRI cases that sought care at the surveillance site over the total number of SRI cases that sought care at any hospital obtained from healthcare utilization surveys (HUS) conducted in the catchment areas of the surveillance sites (data source 2); and *Pop_i_* is the age-specific mid-year population at risk (data source 5).

The equation used to estimate the rates of ILI consultations is provided below [1].

$ILI_{Rate_{i}}=SRI_{Rate_{i}}*Z*X*\frac{Y_{ILI_{i}}}{Y_{SRI_{i}}}$ (2)

Where $ILI_{Rate_{i}}$ is the estimated age-specific rate of ILI outpatient consultations in age group *i*; $SRI_{Rate_{i}}$is the age specific rate of SRI hospitalization (adjusted for non-enrollment and healthcare seeking behavior as obtained in equation 1); *Z* is the proportion of SRI cases that sought outpatient care before hospitalization (data source 1); *X* is ratio of ILI consultation referred to hospital to the total number of ILI consultations obtained from ILI surveillance over the study period (data source 1); $Y_{ILI_{i}}$ is the proportion of ILI cases in age group *i* over the total number of ILI cases after adjusting for non-enrolment (data source 1); $Y_{SRI_{i}}$ is the proportion of SRI cases in age group *i* over the total number of SARI cases after adjusting for non-enrolment (data source 1).

We used the ILI and SRI rates at the sentinel sites as a proxy for the corresponding provinces as previously described [1].

- *Step 2.a: Estimation of ILI and SRI rates in the other provinces*

Estimates of SRI hospitalization rates for the other 7 provinces in South Africa were derived by adjusting the base provinces rates (obtained in Step 1.a) for the provincial-level prevalence of known risk factors for pneumonia obtained from the DHS (data source 3) as previously described (equation 3) [1]. Risk factors included HIV infection, exposure to indoor air pollution, crowding, malnutrition, low birth-weight and non-exclusive breastfeeding [1]. The last three were only included for children aged <5 years. The relative risk of SRI associated with each risk factor was determined from the published literature [1,[[2]](#endnote-2)]. In addition, we adjusted the provincial rates by the proportion of ARI cases seeking care in the given province to the proportion of ARI cases seeking care in the base provinces using data from the DHS (data source 3) as previously described (equation 4) [1]. The healthcare seeking behavior among ARI cases was used as a proxy for SRI cases. An adjustment factor >1 resulted in a greater SRI hospitalization rate in the given province relative to the base province and vice-versa. The equations used for the provincial adjustments are provided below [1].

${Adj}_{Y}=\left( 1+\sum_{i} \left( P_{i,Y}-P_{i,B} \right)\times\left( {RR}_{i}-1 \right) \right)$ (3)

Where ${Adj}_{Y}$ is the adjustment factor for risk factors of SRI for province *Y*; $P_{i,Y}$ is the prevalence of risk factor *i* in province *Y* from the DHS (data source 3); $P_{i,B}$ is the prevalence of risk factor *i* in the base provinces from the DHS (data source 3); and ${RR}_{i}$ is the relative risk of SRI due to risk factor *i*.

${SRI}_{RateH,Y}={SRI}_{RateH,B} \times{Adj}_{Y} \times\frac{{DHS}_{H,Y}}{{DHS}_{H,B}}$ (4)

Where ${SRI}_{RateH,Y}$ is the SRI hospitalization rate in province *Y*; ${SRI}_{RateH,B}$ is the SRI hospitalization rate in the base provinces (obtained in Step 1.a); ${DHS}_{H,Y}$ is the proportion of ARI cases seeking care in province *Y* (from DHS – data source 3); and ${DHS}_{H,B}$ is the proportion of ARI cases seeking care in the base provinces (from DHS - data source 3).

To estimate the ILI rate for the other provinces we used only the adjustment for the healthcare seeking behaviour for ARI.

- *Step 3.a: Estimation of respiratory viruses-associated ILI and SRI rates in all provinces*

We estimated the provincial rates of individual respiratory viruses-associated SRI hospitalization by multiplying the estimated provincial SRI hospitalization rates (obtained in Steps 1.a and 2.a) by the individual respiratory viruses detection rate obtained from sentinel surveillance implemented among inpatients with SRI (data source 1) [1] and adjusting for the attributable fraction (AF) of individual respiratory viruses detection to illness [1]. The equation used to estimate the individual respiratory viruses-associated SRI hospitalization rates is provided below [1].

${ResVirSRI}_{RateH,Y}={SRI}_{RateH,Y} \times{ResVir}\times{ResVir}_{AF}$ (5)

Where ${ResVirSRI}_{RateH,Y}$ is the individual respiratory virus-associated SRI hospitalization rate in province *Y* (including the base provinces); ${SRI}_{RateH,Y}$ is the SRI hospitalization rate in province *Y* (including the base provinces – obtained in Steps 1.a and 2.a); $ResVir$ is the proportion of SRI cases testing positive for individual respiratory viruses (data source 1); and ${ResVir}_{AF}$ if the attributable fraction of the individual respiratory virus detection to illness.

The same approach was used to estimate the individual respiratory viruses-associated ILI, using the ILI specific individual respiratory viruses detection rate and attributable fraction of individual respiratory viruses detection to illness.

- *Step 4.a: Estimation of the number of individual respiratory viruses-associated ILI and SRI in all provinces*

We estimated the provincial number of individual respiratory viruses-associated SRI hospitalizations by multiplying the provincial individual respiratory viruses-associated SRI hospitalization rates (obtained in Step 3.a) by the population at risk (data source 4) in each province over the study period [1]. The equation used to estimate the number of individual respiratory viruses-associated SRI hospitalizations is provided below [1].

${ResVirSRI}_{NumH,Y}={ResVirSRI}_{RateH,Y} \times P{op}_{Y}$ (6)

Where ${ResVirSARI}_{NumH,Y}$ is the number of individual respiratory viruses-associated SRI hospitalizations in province *Y* (including the base provinces); ${ResVirSRI}_{RateH,Y}$ is the individual respiratory viruses-associated SRI hospitalization rate in province *Y* (including the base provinces – obtained in Step 3.a); and $P{op}_{Y}$ is the population in province *Y* (including the base provinces – data source 4).

The same approach was used to estimate the number of individual respiratory viruses-associated ILI.

***Non-medically attended illness***

To estimate the national number and rates of individual respiratory viruses-associated non-medically attended ILI and SRI we used a previously described four-step approach detailed below [1], using HUS data (data source 2) for the 2 syndromes implemented in the catchment areas of the sentinel hospitals/clinics.

- *Step 1.b: Estimation of ILI and SRI rates in the base provinces (North West and KwaZulu-Natal)*

The equation used to estimate the rate of non-hospitalized SRI cases in the base provinces is provided below [1].

${SRI}_{RateNH,B}=\left( \frac{{SRI}_{RateH,B}}{{HUS}_{B}} \right)-{SRI}_{RateH,B}$ (7)

Where ${SRI}_{RateNH,B}$ is the base rate of non-hospitalized SRI; ${SRI}_{RateH,B}$ is the base rate of hospitalized SRI (obtained in Step 1.a); and ${HUS}_{B}$ is the proportion of all SRI cases that are hospitalized in the base provinces from HUSs (data source 2).

The same approach was used to estimate the ILI rates.

- *Step 2.b: Estimation of ILI and SRI rates in the other provinces*

The equation used to estimate the rate of non-hospitalized SRI cases in the other provinces is provided below [1].

${SRI}_{RateNH,Y}={SRI}_{RateNH,B} \times{Adj}_{Y} \times\frac{{DHS}_{NH,Y}}{{DHS}_{NH,B}}$ (8)

Where ${SRI}_{RateNH,Y}$ is the rate of non-hospitalized SRI in province *Y*; ${SRI}_{RateNH,B}$ is the base rate of non-hospitalized SRI (obtained in Step 1.b); ${DHS}_{NH,Y}$ is the proportion of ARI cases not seeking care in province *Y* from the DHS (data source 3);${Adj}_{Y}$ is the adjustment factor for risk factors of SRI for province *Y* (obtained from equation 2.1); and ${DHS}_{NH,B}$ is the proportion of ARI cases not seeking care in the base provinces from the DHS (data source 3).

To estimate the ILI rate for the other provinces we used only the adjustment for the healthcare seeking behaviour for ARI.

- *Step 3.b: Estimation of individual respiratory viruses-associated ILI and SRI rates in all provinces*

The equation used to estimate the non-hospitalized individual respiratory viruses-associated SRI rates is provided below [1].

${ResVirSRI}_{RateNH,Y}={SRI}_{RateNH,Y} \times{ResVir}\times{ResVir}_{AF}$ (9)

Where ${ResVirSRI}_{RateNH,Y}$ is the non-hospitalized individual respiratory viruses-associated SRI rate in province *Y* (including the base provinces); ${SRI}_{RateNH,Y}$ is the non-hospitalized SRI rate in province *Y* (including the base provinces – obtained in Steps 1.b and 2.b); $ResVir$ is the proportion of SRI cases testing positive for individual respiratory viruses (data source 1); and ${ResVir}_{AF}$ if the attributable fraction of individual respiratory viruses detection to illness.

The same approach was used to estimate the individual respiratory viruses-associated ILI, using the ILI specific individual respiratory viruses detection rate and attributable fraction of individual respiratory viruses detection to illness.

- *Step 4.b: Estimation of the number of individual respiratory viruses-associated ILI and SRI in all provinces*

The equation used to estimate the number of non-hospitalized individual respiratory viruses-associated SRI is provided below [1].

${ResVirSRI}_{NumNH,Y}={ResVirSRI}_{RateNH,Y} \times P{op}_{Y}$ (10)

Where ${ResVirSRI}_{NumNH,Y}$ is the number of non-hospitalized individual respiratory viruses-associated SRI cases in province *Y* (including the base provinces); ${ResVirSRI}_{RateNH,Y}$ is the non-hospitalized influenza-associated SRI rate in province Y (including the base provinces – obtained in Step 3.b); and $P{op}_{Y}$ is the population in province Y (including the base provinces – data source 4).

The same approach was used to estimate the number of individual respiratory viruses-associated ILI.

**TABLES**

**Table S1: Positivity proportion of nine respiratory viruses among asymptomatic individuals (controls), outpatients with influenza-like illness (ILI) and inpatients with severe respiratory illness (SRI) by age group, Klerksdorp and Pietermaritzburg, South Africa, 2013-2015**

| **Age**  **(in years)** | **Influenza** | | | **RSV** | | |
| --- | --- | --- | --- | --- | --- | --- |
|  | **Controls**  **% (n/N)** | **ILI**  **% (n/N)** | **SRI**  **% (n/N)** | **Controls**  **% (n/N)** | **ILI**  **% (n/N)** | **SRI**  **% (n/N)** |
| <5 | 1.7 (14/806) | 12.2 (159/1,304) | 5.3 (86/1,632) | 2.5 (20/806) | 9.3 (121/1,304) | 23.2 (379/1,632) |
| 5-44 | 1.4 (13/902) | 15.1 (296/1,958) | 4.1 (69/1,675) | 1.2 (11/902) | 2.9 (57/1,958) | 2.0 (34/1,675) |
| ≥45 | 1.7 (7/411) | 6.1 (25/410) | 4.3 (45/1,051) | 0.7 (3/411) | 2.7 (11/410) | 1.8 (19/1,051) |
| All | 1.6 (34/2,119) | 13.1 (480/3,672) | 4.6 (200/4,358) | 1.6 (34/2,119) | 5.1 (189/3,672) | 9.9 (432/4,358) |
| **Age**  **(in years)** | **Rhinovirus** | | | **HMPV** | | |
|  | **Controls**  **% (n/N)** | **ILI**  **% (n/N)** | **SRI**  **% (n/N)** | **Controls**  **% (n/N)** | **ILI**  **% (n/N)** | **SRI**  **% (n/N)** |
| <5 | 26.4 (213/806) | 33.8 (441/1,304) | 34.1 (557/1,632) | 0.7 (6/806) | 3.5 (46/1,304) | 4.5 (74/1,632) |
| 5-44 | 12.6 (114/902) | 22.7 (444/1,958) | 17.9 (299/1,675) | 0.8 (7/902) | 2.7 (52/1,958) | 0.7 (12/1,675) |
| ≥45 | 6.6 (27/411) | 18.5 (76/410) | 10.1 (106/1,051) | 0.7 (3/411) | 2.0 (8/410) | 1.0 (11/1,051) |
| All | 16.7 (354/2,119) | 26.2 (961/3,672) | 22.1 (962/4,358) | 0.8 (16/2,119) | 2.9 (106/3,672) | 2.2 (97/4,358) |
| **Age**  **(in years)** | **Adenovirus** | | | **Enterovirus** | | |
|  | **Controls**  **% (n/N)** | **ILI**  **% (n/N)** | **SRI**  **% (n/N)** | **Controls**  **% (n/N)** | **ILI**  **% (n/N)** | **SRI**  **% (n/N)** |
| <5 | 14.1 (114/806) | 15.6 (203/1,304) | 17.9 (292/1,632) | 5.7 (46/806) | 7.0 (91/1,304) | 6.9 (112/1,632) |
| 5-44 | 3.3 (30/902) | 5.7 (112/1,958) | 3.9 (66/1,675) | 0.6 (5/902) | 1.6 (31/1,958) | 1.0 (17/1,675) |
| ≥45 | 1.5 (6/411) | 2.9 (12/410) | 2.6 (27/1,051) | 0.0 (0/411) | 0.7 (3/410) | 0.4 (4/1,051) |
| All | 7.1 (150/2,119) | 8.9 (327/3,672) | 8.8 (385/4,358) | 2.4 (51/2,119) | 3.4 (125/3,672) | 3.1 (133/4,358) |
| **Age**  **(in years)** | **PIV types 1-3** | | | **PIV type 1** | | |
|  | **Controls**  **% (n/N)** | **ILI**  **% (n/N)** | **SRI**  **% (n/N)** | **Controls**  **% (n/N)** | **ILI**  **% (n/N)** | **SRI**  **% (n/N)** |
| <5 | 3.3 (27/806) | 7.3 (95/1,304) | 7.9 (129/1,632) | 1.1 (9/806) | 3.0 (39/1,304) | 2.2 (36/1,632) |
| 5-44 | 1.1 (10/902) | 2.8 (54/1,958) | 2.4 (40/1,675) | 0.3 (3/902) | 1.0 (20/1,958) | 1.0 (16/1,675) |
| ≥45 | 0.5 (2/411) | 2.0 (8/410) | 1.7 (18/1,051) | 0.2 (1/411) | 0.7 (3/410) | 0.5 (5/1,051) |
| All | 1.8 (39/2,119) | 4.3 (157/3,672) | 4.3 (187/4,358) | 0.6 (13/2,119) | 1.7 (62/3,672) | 1.3 (57/4,358) |
| **Age**  **(in years)** | **PIV type 2** | | | **PIV type 3** | | |
|  | **Controls**  **% (n/N)** | **ILI**  **% (n/N)** | **SRI**  **% (n/N)** | **Controls**  **% (n/N)** | **ILI**  **% (n/N)** | **SRI**  **% (n/N)** |
| <5 | 0.5 (4/806) | 0.8 (11/1,304) | 0.6 (10/1,632) | 1.7 (14/806) | 3.5 (45/1,304) | 5.3 (86/1,632) |
| 5-44 | 0.2 (2/902) | 0.5 (9/1,958) | 0.6 (10/1,675) | 0.6 (5/902) | 1.3 (26/1,958) | 0.8 (14/1,675) |
| ≥45 | 0.0 (0/411) | 0.7 (3/410) | 0.5 (5/1,051) | 0.2 (1/411) | 0.7 (3/410) | 0.8 (8/1,051) |
| All | 0.3 (6/2,119) | 0.6 (23/3,672) | 0.6 (25/4,358) | 0.9 (20/2,119) | 2.0 (74/3,672) | 2.5 (108/4,358) |

Abbreviations: RSV: respiratory syncytial virus; HMPV: human metapneumovirus; PIV: parainfluenza virus.

**Table S2: National estimates of mean annual number and rate of any medically- and non-medically attended influenza-like illness (ILI) and those associated with seven respiratory viruses in South Africa, 2013-2015**

| **Age** | **Number (95% CI)** | **Rate^a^ (95% CI)** | **Number (95% CI)** | **Rate^a^ (95% CI)** |
| --- | --- | --- | --- | --- |
|  | **Any ILI** | | **Influenza-associated ILI** | |
| <1 | 1,192,483 (1,019,253-1448600) | 102,202.9 (87,356.1-124,153.7) | 79,345 (52,310-112,958) | 6,800.3 (4,483.3-9,681.2) |
| 1-4 | 2,559,895 (2,222,793-3123063) | 56,090.7 (48,704.3-68,430.4) | 347,066 (271,512-453,172) | 7,604.7 (5,949.2-9,929.6) |
| 5-24 | 12,140,762 (10,843,866-15,005,718) | 61,064.6 (54,541.6-75,474.5) | 1,935,727 (1,613,244-2,549,720) | 9,736.2 (8,114.2-12,824.4) |
| 25-44 | 7,822,127 (9,055,108-12,618,399) | 46,560.4 (53,899.6-75,109.7) | 949,698 (1,000,985-1,645,087) | 5,653.0 (5,958.3-9,792.2) |
| 45-64 | 3,221,334 (3,511,480-4,924,890) | 37,377.5 (40,744.0-57,144.0) | 149,436 (97,649-301,535) | 1,733.9 (1,133.0-3,498.7) |
| ≥65 | 836,789 (746,621-1,066,380) | 27,701.7 (24,716.7-35,302.3) | 21,339 (8,564-65,644) | 706.4 (47.3-2,173.1) |
| <5 | 3,752,378 (3,242,045-4,571,663) | 65,479.3 (56,574.0-79,775.9) | 426,411 (323,821-566,130) | 7,440.9 (5,650.7-9,879.0) |
| ≥5 | 24,021,012 (24,157,076-33,615,384) | 49,711.5 (49,993.0-69,567.0) | 3,056,200 (2,711,877-4,561,984) | 6,324.8 (5,612.2-9,441.0) |
| All | 27,773,390 (27,399,120-38,187,048) | 51,383.2 (50,690.8-70,649.4) | 3,482,611 (3,035,697-5,128,114) | 6,443.1 (5,616.3-9,487.5) |
| **Age** | **Number (95% CI)** | **Rate^a^ (95% CI)** | **Number (95% CI)** | **Rate^a^ (95% CI)** |
|  | **RSV-associated ILI** | | **HMPV-associated ILI** | |
| <1 | 93,074 (62,587-130,028) | 7,977.0 (5,364.1-11,144.2) | 28,685 (12,334-47,767) | 2,458.5 (1,057.1-4,093.9) |
| 1-4 | 180,553 (131,041-247,301) | 3,956.2 (2,871.3-5,418.7) | 78,012 (50,735-118,886) | 1,709.3 (1,111.7-2,604.9) |
| 5-24 | 211,089 (117,457-335,936) | 1,061.7 (590.8-1,689.7) | 295,438 (184,045-447,038) | 1,486 (925.7-2,248.5) |
| 25-44 | 159,856 (120,477-322,274) | 951.5 (717.1-1,918.3) | 127,113 (88,048-264,616) | 756.6 (524.1-1,575.1) |
| 45-64 | 60,767 (23,478-153,697) | 705.1 (272.4-1,783.4) | 36,946 (10,451-106,497) | 428.7 (121.3-1,235.7) |
| ≥65 | 22,176 (3,124-67,063) | 734.1 (21.5-2,220.1) | 8,666 (1,235-37,160) | 286.9 (7.3-1,230.2) |
| <5 | 273,627 (193,628-377,329) | 4,774.8 (3,378.8-6,584.4) | 106,697 (63,068-166,652) | 1,861.9 (1,100.5-2,908.1) |
| ≥5 | 453,888 (261,410-878,969) | 939.3 (541.0-1,819.0) | 468,163 (282,542-855,308) | 968.9 (584.7-1,770.1) |
| All | 727,515 (455,038-1,256,296) | 1,346.0 (841.9-2,324.3) | 574,860 (345,609-1,021,960) | 1,063.5 (639.4-1,890.7) |
| **Age** | **Number (95% CI)** | **Rate^a^ (95% CI)** | **Number (95% CI)** | **Rate^a^ (95% CI)** |
|  | **Rhinovirus-associated ILI** | | **Parainfluenza viruses (types 1-3)-associated ILI** | |
| <1 | 195,829 (150,699-255,232) | 16,783.7 (12,915.8-21,874.9) | 68,933 (44,596-99,414) | 5,908.0 (3,822.1-8,520.4) |
| 1-4 | 344,249 (268,491-445,690) | 7,542.9 (5,883.0-9,765.7) | 112,769 (76,784-161,402) | 2,470.9 (1,682.4-3,536.5) |
| 5-24 | 1,866,705 (1,555,244-2,472,705) | 9,389.0 (7,822.4-12,437) | 264,550 (165,443-412,478) | 1,330.6 (832.1-2,074.6) |
| 25-44 | 963,009 (1,013,062-1,666,146) | 5,732.2 (6,030.1-9,917.6) | 134,419 (104,896-275,903) | 800.1 (624.4-1,642.3) |
| 45-64 | 437,146 (393,017-766,171) | 5,072.2 (4,560.2-8,890.0) | 22,031 (7,345-75,305) | 255.6 (83.4-873.8) |
| ≥65 | 96,425 (31,972-188,872) | 3,192.1 (1,058.4-6,252.6) | 12,059 (2,759-48,091) | 399.2 (98.4-1,592.0) |
| <5 | 540,078 (419,190-700,922) | 9,424.4 (7,314.9-12,231.1) | 181,702 (121,379-260,815) | 31,70.7 (2,118.1-4,551.2) |
| ≥5 | 3,363,285 (2,993,293-5,093,892) | 6,960.3 (6,194.6-10,541.8) | 433,059 (270,339-811,776) | 896.2 (559.5-1,680.0) |
| All | 3,903,363 (3,412,483-5,794,814) | 7,221.6 (6,313.4-10,720.9) | 614,761 (391,718-1,072,590) | 1,137.4 (724.7-1,984.4) |
| **Age** | **Number (95% CI)** | **Rate^a^ (95% CI)** | **Number (95% CI)** | **Rate^a^ (95% CI)** |
|  | **Adenovirus-associated ILI** | | **Enterovirus-associated ILI** | |
| <1 | 19,632 (5,465-35,403) | 1,682.6 (468.4-3,034.2) | 35,370 (16,399-56,840) | 3,031.4 (1,405.5-4,871.5) |
| 1-4 | 84,410 (54,505-125,869) | 1,849.5 (1,194.3-2,758.0) | 77,587 (49,972-117,715) | 1,700.0 (1,095-2,579.3) |
| 5-24 | 413,411 (283,039-607,646) | 2,079.3 (1,423.6-3,056.3) | 184,109 (99,464-299,318) | 926.0 (500.3-1,505.5) |
| 25-44 | 176,357 (144,877-353,576) | 1,049.7 (862.4-2,104.6) | 64,315 (32,286-151,882) | 382.8 (192.2-904.1) |
| 45-64 | 37,835 (10,639-108,845) | 439.0 (123.4-1,262.9) | 16,654 (1,630-62,334) | 193.2 (19.7-723.3) |
| ≥65 | 5,648 (937-32,402) | 187 (37.4-1,072.7) | 13,671 (1,396-49,979) | 452.6 (4.3-1,654.5) |
| <5 | 104,042 (59,970-161,272) | 1,815.5 (1,046.5-2,814.2) | 112,957 (66,371-174,555) | 1,971.1 (1,158.2-3,046.0) |
| ≥5 | 633,251 (438,554-1,102,467) | 1,310.5 (907.6-2,281.6) | 278,749 (131,748-563,511) | 576.9 (272.7-1,166.2) |
| All | 737,293 (498,522-1,263,738) | 1,364.1 (922.3-2,338.0) | 391,706 (198,119-738,065) | 724.7 (366.5-1,365.5) |

Abbreviations: CI: confidence intervals; RSV: respiratory syncytial virus; HMPV: human metapneumovirus.

^a^ Rates reported per 100,000 population.

**Table S3: National estimates of mean annual number and rate of any medically attended influenza-like illness (ILI) and those associated with seven respiratory viruses in South Africa, 2013-2015**

| **Age** | **Number (95% CI)** | **Rate^a^ (95% CI)** | **Number (95% CI)** | **Rate^a^ (95% CI)** |
| --- | --- | --- | --- | --- |
|  | **Any ILI** | | **Influenza-associated ILI** | |
| <1 | 272,418 (260,930-283,937) | 23,347.8 (22,363.3-24,335.1) | 18,126 (12,289-24,148) | 1,553.5 (1,053.2-2,069.6) |
| 1-4 | 591,748 (567,893-615,303) | 12,966.0 (12,443.3-13,482.1) | 80,228 (65,671-94,944) | 1,757.9 (1,438.9-2,080.3) |
| 5-24 | 2,892,538 (2,794,345-2,991,247) | 14,548.7 (14,054.8-15,045.1) | 461,188 (394,496-535,409) | 2,319.6 (1,984.2-2,693.0) |
| 25-44 | 2,357,859 (2,275,211-2,443,666) | 14,034.9 (13,543.0-14,545.7) | 286,271 (236,470-338,188) | 1,704.0 (1,407.6-2,013.0) |
| 45-64 | 919,682 (883,862-9,56,469) | 10,671.2 (10,255.5-11,098.0) | 42,664 (22,803-63,943) | 495.0 (264.6-741.9) |
| ≥65 | 198,665 (188,582-208,863) | 6,576.8 (6,243.0-6,914.4) | 5,066 (513-14,281) | 167.7 (16.9-472.8) |
| <5 | 864,166 (828,823-899,240) | 15,079.8 (14,463.0-15,691.8) | 98,354 (77,959-119,092) | 1,716.3 (1,360.4-2,078.2) |
| ≥5 | 6,368,744 (6,142,000-6,600,244) | 13,180.1 (12,710.9-13,659.2) | 795,189 (653,768-951,820) | 1,645.6 (1,353.0-1,969.8) |
| All | 7,232,910 (6,970,823-7,499,484) | 13,381.5 (12,896.6-13,874.7) | 893,543 (731,727-1,070,912) | 1,653.1 (1,353.8-1,981.3) |
| **Age** | **Number (95% CI)** | **Rate^a^ (95% CI)** | **Number (95% CI)** | **Rate^a^ (95% CI)** |
|  | **RSV-associated ILI** | | **HMPV-associated ILI** | |
| <1 | 21,262 (14,895-27,773) | 1,822.3 (1,276.5-2,380.3) | 6,553 (2,895-10,403) | 561.6 (248.1-891.6) |
| 1-4 | 41,737 (31,321-52,558) | 914.5 (686.3-1,151.6) | 18,034 (11,827-25,797) | 395.1 (259.1-565.2) |
| 5-24 | 50,292 (27,200-74,068) | 253.0 (136.8-372.5) | 70,388 (43,155-97,970) | 354.0 (217.1-492.8) |
| 25-44 | 48,186 (28,041-68,886) | 286.8 (166.9-410.0) | 38,317 (19,737-56,868) | 228.1 (117.5-338.5) |
| 45-64 | 17,348 (5,206-33,382) | 201.3 (60.4-387.3) | 10,549 (2,537-23,205) | 122.4 (29.4-269.2) |
| ≥65 | 5,265 (537-14,580) | 174.3 (17.8-482.7) | 2,057 (237-7,428) | 68.1 (6.9-245.9) |
| <5 | 62,999 (46,216-80,331) | 1,099.3 (806.5-1401.8) | 24,587 (14,722-36,199) | 429.0 (256.9-631.7) |
| ≥5 | 121,091 (60,446-190,916) | 250.6 (125.1-395.1) | 121,311 (65,428-185,469) | 251.1 (135.4-383.8) |
| All | 184,090 (106,662-271,246) | 340.6 (197.3-501.8) | 145,898 (80,149-221,668) | 269.9 (148.3-410.1) |
| **Age** | **Number (95% CI)** | **Rate^a^ (95% CI)** | **Number (95% CI)** | **Rate^a^ (95% CI)** |
|  | **Rhinovirus-associated ILI** | | **Parainfluenza viruses (types 1-3)-associated ILI** | |
| <1 | 44,736 (36,061-53,977) | 3,834.1 (3,090.6-4,626.2) | 15,747 (10,405-21,478) | 1,349.6 (891.7-1,840.8) |
| 1-4 | 79,577 (64,849-94,090) | 1,743.6 (1,420.9-2,061.6) | 26,068 (18,207-35,265) | 571.2 (398.9-772.7) |
| 5-24 | 444,743 (376,899-516,137) | 2,236.9 (1,895.7-2,596.0) | 63,029 (38,659-89,437) | 317.0 (194.4-449.8) |
| 25-44 | 290,283 (239,513-343,281) | 1,727.9 (1,425.7-2,043.3) | 40,518 (23,749-60,000) | 241.2 (141.4-357.1) |
| 45-64 | 124,805 (91,584-158,881) | 1,448.1 (1062.7-1,843.5) | 6,290 (654-15,813) | 73.0 (7.1-183.5) |
| ≥65 | 22,894 (7,123-41,192) | 757.9 (235.8-1,363.6) | 2,862 (273-10,736) | 94.7 (9.3-355.4) |
| <5 | 124,313 (100,910-148,067) | 2,169.3 (1,760.9-2,583.8) | 41,815 (28,611-56,743) | 729.7 (499.3-990.2) |
| ≥5 | 882,725 (715,118-1,059,490) | 1,826.8 (1,479.9-2,192.6) | 112,699 (62,408-175,986) | 233.2 (129.2-364.2) |
| All | 1,007,038 (816,028-1,207,557) | 1,863.1 (1,509.7-2,234.1) | 154,514 (91,019-232,728) | 285.9 (168.4-430.6) |
| **Age** | **Number (95% CI)** | **Rate^a^ (95% CI)** | **Number (95% CI)** | **Rate^a^ (95% CI)** |
|  | **Adenovirus-associated ILI** | | **Enterovirus-associated ILI** | |
| <1 | 4,485 (1,186-7,665) | 384.4 (101.6-656.9) | 8,080 (3,601-12,244) | 692.5 (308.6-1049.3) |
| 1-4 | 19,512 (13,000-27,437) | 427.5 (284.8-601.2) | 17,936 (11,700-25,684) | 393.0 (256.4-562.8) |
| 5-24 | 98,495 (66,659-130,354) | 495.4 (335.3-655.6) | 43,864 (23,431-66,049) | 220.6 (117.8-332.2) |
| 25-44 | 53,161 (33,251-75,984) | 316.4 (197.9-452.3) | 19,386 (7,179-33,470) | 115.4 (42.7-199.2) |
| 45-64 | 10,800 (2,543-23,444) | 125.3 (29.5-272.0) | 4,754 (493-13,166) | 55.2 (5.3-152.8) |
| ≥65 | 1,341 (142-7,192) | 44.4 (4.7-238.1) | 3,245 (311-10,897) | 107.4 (10.2-360.7) |
| <5 | 23,997 (14,186-35,102) | 418.7 (247.5-612.5) | 26,016 (15,301-37,928) | 454.0 (267.0-661.8) |
| ≥5 | 163,797 (102,452-236,973) | 339.0 (212.0-490.4) | 71,249 (30,609-123,581) | 147.4 (63.3-255.8) |
| All | 187,794 (116,637-272,074) | 347.4 (215.8-503.4) | 97,265 (45,910-161,509) | 179.9 (84.9-298.8) |

Abbreviations: CI: confidence intervals; RSV: respiratory syncytial virus; HMPV: human metapneumovirus.

^a^ Rates reported per 100,000 population.

**Table S4: National estimates of mean annual number and rate of any non-medically attended influenza-like illness (ILI) and those associated with seven respiratory viruses in South Africa, 2013-2015**

| **Age** | **Number (95% CI)** | **Rate^a^ (95% CI)** | **Number (95% CI)** | **Rate^a^ (95% CI)** |
| --- | --- | --- | --- | --- |
|  | **Any ILI** | | **Influenza-associated ILI** | |
| <1 | 920,065 (758,323-1,164,663) | 78,855.1 (64,992.8-9,9818.6) | 61,219 (40,021-88,810) | 5,246.8 (3,430.0-7,611.5) |
| 1-4 | 1,968,147 (1,654,900-2,507,760) | 43,124.7 (36,261-54,948.3) | 266,838 (205,841-358,228) | 5,846.8 (4,510.2-7,849.2) |
| 5-24 | 9,248,224 (8,049,521-12,014,471) | 46,516.0 (40,486.8-60,429.4) | 1,474,539 (1,218,748-2,014,311) | 7,416.5 (6,130.0-10,131.4) |
| 25-44 | 5,464,268 (6,779,897-10,174,733) | 32,525.5 (40,356.6-60,564.0) | 663,427 (764,515-1,306,899) | 3,949.0 (4,550.7-7,779.2) |
| 45-64 | 2,301,652 (2,627,618-3,968,421) | 26,706.3 (30,488.5-46,046.0) | 106,772 (74,846-237,592) | 1,238.9 (868.4-2,756.8) |
| ≥65 | 638,124 (558,039-857,517) | 21,125.0 (18,473.8-28,387.9) | 16,273 (,1680-51,363) | 538.7 (54.1-1,700.4) |
| <5 | 2,888,212 (2,413,222-3,672,423) | 50,399.5 (42,110.9-64,084.1) | 328,057 (245,862-447,038) | 5,724.6 (4,290.3-7,800.8) |
| ≥5 | 17,652,268 (18,015,075-27,015,141) | 36,531.3 (37,282.2-55,907.8) | 2,261,011 (2,058,109-3,610,164) | 4,679.2 (4,259.3-7,471.2) |
| All | 20,540,480 (20,428,297-30,687,564) | 38,001.7 (37,794.1-56,774.7) | 2,589,068 (2,303,970-4,057,202) | 4,790.0 (4,262.5-7,506.2) |
| **Age** | **Number (95% CI)** | **Rate^a^ (95% CI)** | **Number (95% CI)** | **Rate^a^ (95% CI)** |
|  | **RSV-associated ILI** | | **HMPV-associated ILI** | |
| <1 | 71,812 (47,692-102,255) | 6,154.7 (4,087.5-8,763.8) | 22,132 (9,439-37,364) | 1,896.8 (808.9-3,202.3) |
| 1-4 | 138,816 (99,720-194,743) | 3,041.6 (2,185.0-4,267.1) | 59,978 (38,908-93,089) | 1,314.2 (852.5-2,039.7) |
| 5-24 | 160,797 (90,257-261,868) | 808.8 (454-1,317.1) | 225,050 (140,890-349,068) | 1,131.9 (708.6-1,755.7) |
| 25-44 | 111,670 (92,436-253,388) | 664.7 (550.2-1,508.3) | 88,796 (68,311-207,748) | 528.5 (406.6-1,236.6) |
| 45-64 | 43,419 (18,272-120,315) | 503.8 (212.0-1,396.0) | 26,397 (7,914-83,292) | 306.3 (91.8-966.4) |
| ≥65 | 16,911 (1,703-52,483) | 559.8 (56.1-1,737.4) | 6,609 (658-29,732) | 218.8 (20.8-984.3) |
| <5 | 210,628 (147,412-296,998) | 3,675.5 (2,572.4-5,182.6) | 82,110 (48,346-130,453) | 1,432.8 (843.6-2,276.4) |
| ≥5 | 332,797 (200,964-688,053) | 688.7 (415.9-1,423.9) | 346,852 (217,114-669,839) | 717.8 (449.3-1,386.2) |
| All | 543,425 (348,376-985,050) | 1,005.4 (644.5-1,822.4) | 428,962 (265,460-800,292) | 793.6 (491.1-1,480.6) |
| **Age** | **Number (95% CI)** | **Rate^a^ (95% CI)** | **Number (95% CI)** | **Rate^a^ (95% CI)** |
|  | **Rhinovirus-associated ILI** | | **Parainfluenza viruses (types 1-3)-associated ILI** | |
| <1 | 151,093 (114,638-201,255) | 12,949.6 (9,825.1-17,248.7) | 53,186 (34,191-77,936) | 4,558.4 (2,930.4-6,679.5) |
| 1-4 | 264,672 (203,642-351,600) | 5,799.3 (4,462.1-7,704.0) | 86,701 (58,577-126,137) | 1,899.7 (1,283.5-2,763.8) |
| 5-24 | 1,421,962 (1,178,345-1,956,568) | 7,152.1 (5,926.7-9,841.0) | 201,521 (126,784-323,041) | 1,013.6 (637.7-1,624.8) |
| 25-44 | 672,726 (773,549-1,322,865) | 4,004.3 (4,604.5-7,874.2) | 93,901 (81,147-215,903) | 558.9 (483.0-1,285.1) |
| 45-64 | 312,341 (301,433-607,290) | 3,624.1 (3,497.6-7,046.4) | 15,741 (1,555-59,492) | 182.6 (18.7-690.3) |
| ≥65 | 73,531 (24,849-147,680) | 2,434.2 (822.6-4,888.9) | 9,197 (937-37,355) | 304.5 (31.0-1,236.6) |
| <5 | 415,765 (318,280-552,855) | 7,255.1 (5,554.0-9,647.4) | 139,887 (92,768-204,072) | 2,441.0 (1,618.8-3,561.1) |
| ≥5 | 2,480,560 (2,278,175-4,034,402) | 5,133.5 (4,714.7-8,349.2) | 320,360 (207,931-635,790) | 663.0 (430.3-1,315.8) |
| All | 2,896,325 (2,596,455-4,587,257) | 5,358.5 (4,803.7-8,486.8) | 460,247 (300,699-839,862) | 851.5 (556.3-1,553.8) |
| **Age** | **Number (95% CI)** | **Rate^a^ (95% CI)** | **Number (95% CI)** | **Rate^a^ (95% CI)** |
|  | **Adenovirus-associated ILI** | | **Enterovirus-associated ILI** | |
| <1 | 15,147 (4,279-27,738) | 1,298.2 (366.7-2,377.3) | 27,290 (12,798-44,596) | 2,338.9 (1,096.9-3,822.1) |
| 1-4 | 64,898 (41,505-98,432) | 1,422.0 (909.4-2,156.8) | 59,651 (38,272-92,031) | 1,307.0 (838.6-2,016.5) |
| 5-24 | 314,916 (216,380-477,292) | 1,583.9 (1,088.3-2,400.6) | 140,245 (76,033-233,269) | 705.4 (382.4-1,173.3) |
| 25-44 | 123,196 (111,626-277,592) | 733.3 (664.4-1,652.3) | 44,929 (25,107-118,412) | 267.4 (149.4-704.8) |
| 45-64 | 27,035 (8,096-85,401) | 313.7 (93.9-990.9) | 11,900 (1,237-49,168) | 138.1 (13.2-570.5) |
| ≥65 | 4,307 (444-25,210) | 142.6 (14.6-834.6) | 10,426 (1,039-39,082) | 345.2 (35.2-1,293.8) |
| <5 | 80,045 (45,784-126,170) | 1,396.8 (798.9-2,201.7) | 86,941 (51,070-136,627) | 1,517.1 (891.2-2,384.1) |
| ≥5 | 469,454 (33,6102-865,494) | 971.5 (695.6-1,791.1) | 207,500 (101,139-439,930) | 429.4 (209.3-910.4) |
| All | 549,499 (381,885-991,664) | 1,016.6 (706.5-1,834.7) | 294,441 (152,209-576,556) | 544.7 (281.6-1,066.7) |

Abbreviations: CI: confidence intervals; RSV: respiratory syncytial virus; HMPV: human metapneumovirus.

^a^ Rates reported per 100,000 population.

**Table S5: National estimates of mean annual number and rate of any medically- and non-medically attended severe respiratory illness (SRI) and those associated with seven respiratory viruses in South Africa, 2013-2015**

| **Age** | **Number (95% CI)** | **Rate^a^ (95% CI)** | **Number (95% CI)** | **Rate^a^ (95% CI)** |
| --- | --- | --- | --- | --- |
|  | **Any SRI** | | **Influenza-associated SRI** | |
| <1 | 353,164 (298,084-420,787) | 30,268.3 (25,547.6-36,064.0) | 10,635 (7,007-14,994) | 911.5 (600.5-1,285.1) |
| 1-4 | 210,759 (176,396-255,417) | 4,618.0 (3,865.1-5,596.5) | 12,266 (8,120-17,383) | 268.8 (177.9-380.9) |
| 5-24 | 254,972 (214,447-302,452) | 1,282.4 (1,078.6-1,521.2) | 7,767 (3,153-13,770) | 39.1 (15.9-69.3) |
| 25-44 | 643,113 (549,666-761,989) | 3,828.1 (3,271.8-4,535.7) | 18,884 (12,546-26,822) | 112.4 (74.7-159.7) |
| 45-64 | 484,644 (414,934-580,786) | 5,623.4 (4,814.5-6,738.9) | 11,879 (6,729-17,888) | 137.8 (78.1-207.6) |
| ≥65 | 321,473 (268,891-386245) | 10,642.3 (8,901.6-12,786.6) | 9,255 (2,915-17,276) | 306.4 (96.5-571.9) |
| <5 | 563,923 (474,479-676,202) | 9,840.5 (8,279.7-11,799.8) | 22,901 (15,126-32,377) | 399.6 (263.9-565.0) |
| ≥5 | 1,704,202 (1,447,937-2,031,470) | 3,526.8 (2,996.5-4,204.1) | 47,785 (25,341-75,756) | 98.9 (52.4-156.8) |
| All | 2,268,125 (1,922,416-2,707,672) | 4,196.2 (3,556.6-5,009.4) | 70,686 (40,467-108,131) | 130.8 (74.9-200.1) |
| **Age** | **Number (95% CI)** | **Rate^a^ (95% CI)** | **Number (95% CI)** | **Rate^a^ (95% CI)** |
|  | **RSV-associated SRI** | | **HMPV-associated SRI** | |
| <1 | 90,650 (73,430-113,488) | 7,769.2 (6,293.4-9,726.6) | 13,753 (9,477-18,889) | 1,178.7 (812.2-1,618.9) |
| 1-4 | 25,482 (18,462-33,968) | 558.3 (4,04.5-744.3) | 7,825 (4,643-11,801) | 171.5 (101.7-258.6) |
| 5-24 | 3,330 (706-6,994) | 16.7 (3.6-35.2) | 267 (22-1,649) | 1.3 (0.1-8.3) |
| 25-44 | 4,946 (2,239-8,344) | 29.4 (13.3-49.7) | 827 (59-2,378) | 4.9 (0.4-14.2) |
| 45-64 | 3,846 (1,140-7,447) | 44.6 (13.2-86.4) | 853 (43-2,458) | 9.9 (0.5-28.5) |
| ≥65 | 5,403 (1,229-12,296) | 178.9 (40.7-407.1) | 1,715 (29-5,523) | 56.8 (1.0-182.8) |
| <5 | 116,132 (91,892-147,456) | 2,026.5 (1,603.5-2573.1) | 21,578 (14,120-30,689) | 376.5 (246.4-535.5) |
| ≥5 | 17,525 (5,312-35,079) | 36.3 (11.0-72.6) | 3,662 (153-12,007) | 7.6 (0.3-24.8) |
| All | 133,657 (97,203-182,535) | 247.3 (179.8-337.7) | 25,240 (14,273-42,696) | 46.7 (26.4-79.0) |
| **Age** | **Number (95% CI)** | **Rate^a^ (95% CI)** | **Number (95% CI)** | **Rate^a^ (95% CI)** |
|  | **Rhinovirus-associated SRI** | | **Parainfluenza viruses (types 1-3)-associated SRI** | |
| <1 | 52,795 (41,564-67,143) | 4,524.8 (3,562.3-5,754.6) | 16,896 (11,904-22869) | 1,448.1 (1,020.2-1,960.0) |
| 1-4 | 41,082 (31,902-52,833) | 900.2 (699.0-1,157.6) | 11,608 (7,844-16,802) | 254.3 (171.9-368.2) |
| 5-24 | 38,811 (26,953-52,082) | 195.2 (135.6-262.0) | 3,918 (774-8,124) | 19.7 (3.9-40.9) |
| 25-44 | 54,900 (42,339-70,113) | 326.8 (252.0-417.3) | 7,274 (3,747-11,637) | 43.3 (22.3-69.3) |
| 45-64 | 18,884 (12,252-27,845) | 219.1 (142.2-323.1) | 3,413 (1,083-6,578) | 39.6 (12.6-76.3) |
| ≥65 | 9,928 (2,893-19,203) | 328.7 (95.8-635.7) | 4,569 (34-10,202) | 151.3 (1.1-337.7) |
| <5 | 93,877 (73,466-119,976) | 1,638.2 (1,282.0-2,093.6) | 28,504 (19,746-39,671) | 497.4 (344.6-692.3) |
| ≥5 | 122,523 (84,435-169,241) | 253.6 (174.7-350.2) | 19,174 (5,636-36,539) | 39.7 (11.7-75.6) |
| All | 216,400 (157,900-289,217) | 400.4 (292.1-535.1) | 47,678 (25,382-76,210) | 88.2 (47.0-141.0) |
| **Age** | **Number (95% CI)** | **Rate^a^ (95% CI)** | **Number (95% CI)** | **Rate^a^ (95% CI)** |
|  | **Adenovirus-associated SRI** | | **Enterovirus-associated SRI** | |
| <1 | 16,183 (11,506-22,070) | 1,387.0 (986.1-1,891.5) | 8,544 (5,207-12,397) | 732.3 (446.3-1,062.5) |
| 1-4 | 22,089 (16,092-29,573) | 484.0 (352.6-648.0) | 8,666 (5,299-12,903) | 189.9 (116.1-282.7) |
| 5-24 | 2,735 (27-6,311) | 13.8 (0.1-31.7) | 4,327 (1,250-8,852) | 21.8 (6.3-44.5) |
| 25-44 | 7,619 (4,204-12,183) | 45.4 (25-72.5) | 3,825 (1,340-6,971) | 22.8 (8.0-41.5) |
| 45-64 | 3,424 (1,083-6,597) | 39.7 (12.6-76.5) | 975 (43-2,609) | 11.3 (0.5-30.3) |
| ≥65 | 2,886 (31-7,402) | 95.5 (1.0-245.0) | 2,349 (30-6,306) | 77.8 (1.0-208.8) |
| <5 | 38,272 (27,597-51,643) | 667.8 (481.6-901.2) | 17,210 (10,506-25,299) | 300.3 (183.3-441.5) |
| ≥5 | 16,664 (5,344-32,491) | 34.5 (11.1-67.2) | 11,476 (2,663-24,735) | 23.7 (5.5-51.2) |
| All | 54,936 (32,941-84,132) | 101.6 (60.9-155.7) | 28,686 (13,169-50,034) | 53.1 (24.4-92.6) |

Abbreviations: CI: confidence intervals; RSV: respiratory syncytial virus; HMPV: human metapneumovirus.

^a^ Rates reported per 100,000 population.

**Table S6: National estimates of mean annual number and rate of any medically attended severe respiratory illness (SRI) and those associated with seven respiratory viruses in South Africa, 2013-2015**

| **Age** | **Number (95% CI)** | **Rate^a^ (95% CI)** | **Number (95% CI)** | **Rate^a^ (95% CI)** |
| --- | --- | --- | --- | --- |
|  | **Any SRI** | | **Influenza-associated SRI** | |
| <1 | 163,084 (154,501-172,155) | 13,977.3 (13,241.6-14,754.7) | 4,910 (3,391-6,545) | 420.8 (290.6-560.9) |
| 1-4 | 97,476 (91,703-103,328) | 2,135.8 (2,009.3-2,264.0) | 5,673 (3,897-7,572) | 124.3 (85.4-165.9) |
| 5-24 | 118,366 (112,448-124,254) | 595.3 (565.6-625.0) | 3,606 (1,462-6,040) | 18.1 (7.4-30.4) |
| 25-44 | 300,373 (288,178-312,902) | 1,787.9 (1,715.3-1,862.5) | 8,820 (6,162-11,788) | 52.5 (36.7-70.2) |
| 45-64 | 224,513 (214,741-234,672) | 2,605.0 (2,491.7-2,722.9) | 5,503 (3,216-7,860) | 63.9 (37.3-91.2) |
| ≥65 | 147,889 (139,856-156,128) | 4,895.8 (4,629.9-5,168.6) | 4,257 (1,313-7,714) | 140.9 (43.5-255.4) |
| <5 | 260,560 (246,203-275,482) | 4,546.8 (4,296.3-4,807.2) | 10,583 (7,288-14,117) | 184.7 (127.2-246.3) |
| ≥5 | 791,141 (755,223-827,955) | 1,637.3 (1,562.9-1,713.5) | 22,186 (12,152-33,402) | 45.9 (25.1-69.1) |
| All | 1,051,701 (1,001,426-1,103,437) | 1,945.7 (1,852.7-2,041.5) | 32,769 (19,440-47,518) | 60.6 (36.0-87.9) |
| **Age** | **Number (95% CI)** | **Rate^a^ (95% CI)** | **Number (95% CI)** | **Rate^a^ (95% CI)** |
|  | **RSV-associated SRI** | | **HMPV-associated SRI** | |
| <1 | 41,860 (37,229-46,975) | 3,587.7 (,3190.7-4,026) | 6,352 (4,566-8,204) | 544.4 (391.3-703.1) |
| 1-4 | 11,785 (9,174-14,576) | 258.2 (201.0-319.4) | 3,619 (2,224-5,178) | 79.3 (48.7-113.5) |
| 5-24 | 1,545 (348-3,216) | 7.8 (1.8-16.2) | 124 (11-724) | 0.6 (0.1-3.6) |
| 25-44 | 2,311 (1,099-3,774) | 13.8 (6.5-22.5) | 386 (30-1,108) | 2.3 (0.2-6.6) |
| 45-64 | 1,782 (541-3,312) | 20.7 (6.3-38.4) | 395 (21-1,098) | 4.6 (0.2-12.7) |
| ≥65 | 2,486 (623-5,477) | 82.3 (20.6-181.3) | 789 (15-2,561) | 26.1 (0.5-84.8) |
| <5 | 53,645 (46,403-61,551) | 936.1 (809.7-1,074.1) | 9,971 (6,790-13,382) | 174.0 (118.5-233.5) |
| ≥5 | 8,124 (2,610-15,778) | 16.8 (5.4-32.7) | 1,694 (77-5,491) | 3.5 (0.2-11.4) |
| All | 61,769 (49,013-77,329) | 114.3 (90.7-143.1) | 11,665 (6,867-18,873) | 21.6 (12.7-34.9) |
| **Age** | **Number (95% CI)** | **Rate^a^ (95% CI)** | **Number (95% CI)** | **Rate^a^ (95% CI)** |
|  | **Rhinovirus-associated SRI** | | **Parainfluenza viruses (types 1-3)-associated SRI** | |
| <1 | 24,380 (20,830-28,055) | 2,089.5 (1,785.3-2,404.5) | 7,802 (5,836-9,822) | 668.7 (500.1-841.8) |
| 1-4 | 19,000 (15,936-22,311) | 416.3 (349.2-488.9) | 5,368 (3,796-7,305) | 117.6 (83.2-160.1) |
| 5-24 | 18,017 (13,207-22,504) | 90.6 (66.4-113.2) | 1,818 (362-3,595) | 9.1 (1.8-18.1) |
| 25-44 | 25,642 (21,289-30,168) | 152.6 (126.7-179.6) | 3,397 (1,788-5,297) | 20.2 (10.6-31.5) |
| 45-64 | 8,748 (5,910-12,110) | 101.5 (68.6-140.5) | 1,582 (531-2,962) | 18.4 (6.2-34.4) |
| ≥65 | 4,568 (1,330-8,560) | 151.2 (44.0-283.4) | 2,102 (15-4,585) | 69.6 (0.5-151.8) |
| <5 | 43,380 (36,766-50,366) | 757.0 (641.6-878.9) | 13,170 (9,631-17,127) | 229.8 (168.1-298.9) |
| ≥5 | 56,975 (41,735-73,341) | 117.9 (86.4-151.8) | 8,899 (2,695-16,438) | 18.4 (5.6-34.0) |
| All | 100,355 (78,500-123,707) | 185.7 (145.2-228.9) | 22,069 (12,326-33,565) | 40.8 (22.8-62.1) |
| **Age** | **Number (95% CI)** | **Rate^a^ (95% CI)** | **Number (95% CI)** | **Rate^a^ (95% CI)** |
|  | **Adenovirus-associated SRI** | | **Enterovirus-associated SRI** | |
| <1 | 7,473 (5,645-9,513) | 640.5 (483.8-815.3) | 3,945 (2,511-5,388) | 338.1 (215.2-461.8) |
| 1-4 | 10,216 (7,928-12,696) | 223.8 (173.7-278.2) | 4,009 (2,546-5,629) | 87.8 (55.8-123.3) |
| 5-24 | 1,269 (13-2,831) | 6.4 (0.1-14.2) | 2,009 (677-3,911) | 10.1 (3.4-19.7) |
| 25-44 | 3,559 (2,015-5,456) | 21.2 (12.0-32.5) | 1,786 (664-3,156) | 10.6 (4.0-18.8) |
| 45-64 | 1,586 (531-2,962) | 18.4 (6.2-34.4) | 451 (21-1,137) | 5.2 (0.2-13.2) |
| ≥65 | 1,329 (15-3,315) | 44.0 (0.5-109.7) | 1,080 (15-2,762) | 35.8 (0.5-91.4) |
| <5 | 17,689 (13,572-22,209) | 308.7 (236.8-387.5) | 7,954 (5,057-11,017) | 138.8 (88.2-192.2) |
| ≥5 | 7,743 (2,574-14,563) | 16.0 (5.3-30.1) | 5,326 (1,377-10,964) | 11.0 (2.8-22.7) |
| All | 25,432 (16,146-36,771) | 47.1 (29.9-68.0) | 13,280 (6,434-21,981) | 24.6 (11.9-40.7) |

Abbreviations: CI: confidence intervals; RSV: respiratory syncytial virus; HMPV: human metapneumovirus.

^a^ Rates reported per 100,000 population.

**Table S7: National estimates of mean annual number and rate of any non-medically attended severe respiratory illness (SRI) and those associated with seven respiratory viruses in South Africa, 2013-2015**

| **Age** | **Number (95% CI)** | **Rate^a^ (95% CI)** | **Number (95% CI)** | **Rate^a^ (95% CI)** |
| --- | --- | --- | --- | --- |
|  | **Any SRI** | | **Influenza-associated SRI** | |
| <1 | 190,080 (143,583-248,632) | 16,291.0 (12,305.9-21,309.2) | 5,725 (3,616-8,449) | 490.7 (309.9-724.1) |
| 1-4 | 113,283 (84,693-152,089) | 2,482.2 (1,855.7-3,332.5) | 6,593 (4,223-9,811) | 144.5 (92.5-215.0) |
| 5-24 | 136,606 (101,999-178,198) | 687.1 (513.0-896.3) | 4,161 (1,691-7,730) | 20.9 (8.5-38.9) |
| 25-44 | 342,740 (261,488-449,087) | 2,040.1 (1556.5-2,673.1) | 10,064 (6,384-15,034) | 59.9 (38.0-89.5) |
| 45-64 | 260,131 (200,193-346,114) | 3,018.3 (2,322.9-4,016.0) | 6,376 (3,513-10,028) | 74.0 (40.8-116.4) |
| ≥65 | 173,584 (129,035-230,117) | 5,746.5 (4,271.7-7,618.0) | 4,998 (1,602-9,562) | 165.5 (53.0-316.5) |
| <5 | 303,363 (228,276-400,720) | 5,293.7 (3,983.4-6,992.6) | 12,318 (7,838-18,260) | 215.0 (136.8-318.6) |
| ≥5 | 913,061 (692,714-1,203,515) | 1,889.6 (1,433.6-2,490.7) | 25,599 (13,189-42,354) | 5.0 (27.3-87.7) |
| All | 1,216,424 (920,990-1,604,235) | 2,250.5 (1,703.9-2,968.0) | 37,917 (21,027-60,613) | 70.1 (38.9-112.1) |
| **Age** | **Number (95% CI)** | **Rate^a^ (95% CI)** | **Number (95% CI)** | **Rate^a^ (95% CI)** |
|  | **RSV-associated SRI** | | **HMPV-associated SRI** | |
| <1 | 48,790 (36,201-66,513) | 4,181.6 (3,102.6-5,700.6) | 7,401 (4,911-10,685) | 634.3 (420.9-915.7) |
| 1-4 | 13,697 (9,288-19,392) | 300.1 (203.5-424.9) | 4,206 (2,419-6,623) | 92.2 (53.0-145.1) |
| 5-24 | 1,785 (358-3,778) | 9.0 (1.8-19.0) | 143 (11-925) | 0.7 (0.1-4.6) |
| 25-44 | 2,635 (1,140-4,570) | 15.7 (6.8-27.2) | 441 (29-1,270) | 2.6 (0.2-7.6) |
| 45-64 | 2,064 (599-4,135) | 23.9 (6.9-48.0) | 458 (22-1,360) | 5.3 (0.3-15.8) |
| ≥65 | 2,917 (606-6,819) | 96.6 (20.0-225.7) | 926 (14-2,962) | 30.7 (0.5-98.0) |
| <5 | 62,487 (45,489-85,905) | 1,090.4 (793.8-1,499.0) | 11,607 (7,330-17,307) | 202.5 (127.9-302.0) |
| ≥5 | 9,401 (2,702-19,301) | 19.5 (5.6-39.9) | 1,968 (76-6,516) | 4.1 (0.2-13.5) |
| All | 71,888 (48,190-105,206) | 133.0 (89.2-194.6) | 13,575 (7,406-23,823) | 25.1 (13.7-44.1) |
| **Age** | **Number (95% CI)** | **Rate^a^ (95% CI)** | **Number (95% CI)** | **Rate^a^ (95% CI)** |
|  | **Rhinovirus-associated SRI** | | **Parainfluenza viruses (types 1-3)-associated SRI** | |
| <1 | 28,415 (20,734-39,088) | 2,435.3 (1,777.0-3,350.0) | 9,094 (6,068-13,047) | 779.4 (520-1118.2) |
| 1-4 | 22,082 (15,966-30,522) | 483.8 (349.8-668.8) | 6,240 (4,048-9,497) | 136.7 (88.7-208.1) |
| 5-24 | 20,794 (13,746-29,578) | 104.6 (69.1-148.8) | 2,100 (412-4,529) | 10.6 (2.1-22.8) |
| 25-44 | 29,258 (21,050-39,945) | 174.2 (125.3-237.8) | 3,877 (1,959-6,340) | 23.1 (11.7-37.7) |
| 45-64 | 10,136 (6,342-15,735) | 117.6 (73.6-182.6) | 1,831 (552-3,616) | 21.2 (6.4-42.0) |
| ≥65 | 5,360 (1,563-10,643) | 177.4 (51.7-352.3) | 2,467 (19-5,617) | 81.7 (0.6-185.9) |
| <5 | 50,497 (36,700-69,610) | 881.2 (640.4-1214.7) | 15,334 (10,115-22,544) | 267.6 (176.5-393.4) |
| ≥5 | 65,548 (42,700-95,900) | 135.7 (88.4-198.5) | 10,275 (2,941-20,101) | 21.3 (6.1-41.6) |
| All | 116,045 (79,400-165,510) | 214.7 (146.9-306.2) | 25,609 (13,056-42,645) | 47.4 (24.2-78.9) |
| **Age** | **Number (95% CI)** | **Rate^a^ (95% CI)** | **Number (95% CI)** | **Rate^a^ (95% CI)** |
|  | **Adenovirus-associated SRI** | | **Enterovirus-associated SRI** | |
| <1 | 8,710 (5,861-12,557) | 746.5 (502.3-1,076.2) | 4,599 (2,696-7,009) | 394.2 (231.0-600.7) |
| 1-4 | 11,873 (8,164-16,877) | 260.2 (178.9-369.8) | 4,657 (2,753-7,274) | 102.0 (60.3-159.4) |
| 5-24 | 1,466 (14-3,480) | 7.4 (0.1-17.5) | 2,318 (573-4,941) | 11.7 (2.9-24.9) |
| 25-44 | 4,060 (2,189-6,727) | 24.2 (13.0-40.0) | 2,039 (676-3,815) | 12.1 (4.0-22.7) |
| 45-64 | 1,838 (552-3,635) | 21.3 (6.4-42.2) | 524 (22-1,472) | 6.1 (0.3-17.1) |
| ≥65 | 1,557 (16-4,087) | 51.5 (0.5-135.3) | 1,269 (15-3,544) | 42.0 (0.5-117.3) |
| <5 | 20,583 (14,025-29,434) | 359.2 (244.7-513.6) | 9,256 (5,449-14,282) | 161.5 (95.1-249.2) |
| ≥5 | 8,921 (2,770-17,928) | 18.5 (5.7-37.1) | 6,150 (1,286-13,771) | 12.7 (2.7-28.5) |
| All | 29,504 (16,795-47,361) | 54.6 (31.1-87.6) | 15,406 (6,735-28,053) | 28.5 (12.5-51.9) |

Abbreviations: CI: confidence intervals; RSV: respiratory syncytial virus; HMPV: human metapneumovirus.

^a^ Rates reported per 100,000 population.

**Table S8: National estimates of mean annual number and rate of parainfluenza virus types 1-3-associated influenza-like illness (ILI) in South Africa, 2013-2015**

| **Age** | **Parainfluenza virus type 1-associated ILI** | | | | | |
| --- | --- | --- | --- | --- | --- | --- |
|  | **Total** | | **Medically attended** | | **Non-medically attended** | |
|  | **Number (95% CI)** | **Rate^a^ (95% CI)** | **Number (95% CI)** | **Rate^a^ (95% CI)** | **Number (95% CI)** | **Rate^a^ (95% CI)** |
| <1 | 18,789 (5,390-34,133) | 1,610.3 (462.0-2,925.4) | 4,292 (1,183-7,495) | 367.8 (101.3-642.4) | 14,497 (4,207-26,638) | 1242.5 (360.6-2,283) |
| 1-4 | 56,900 (33,535-90,496) | 1,246.8 (734.8-1,982.9) | 13,154 (7,784-19,813) | 288.2 (170.5-434.1) | 43,746 (25,751-70,683) | 958.5 (564.2-1,548.7) |
| 5-24 | 97,687 (37,047-177,061) | 491.3 (186.3-890.6) | 23,275 (8817-39,326) | 117.1 (44.3-197.8) | 74,412 (28,230-137,735) | 374.3 (142.0-692.8) |
| 25-44 | 50,765 (20,644-126,317) | 302.2 (122.9-751.9) | 15,302 (4,752-28,028) | 91.1 (28.3-166.8) | 35,463 (15,892-98,289) | 211.1 (94.6-585.1) |
| 45-64 | 11,628 (1,201-49,555) | 134.9 (13.7-575.0) | 3,319 (347-10,506) | 38.5 (38.3-121.9) | 8,309 (837-39,049) | 96.4 (9.7-453.1) |
| ≥65 | 9,548 (974-43,395) | 316.1 (31.3-1,436.6) | 2,267 (231-10,358) | 75.0 (74.8-342.9) | 7,281 (741-33,037) | 241.0 (23.8-1,093.7) |
| <5 | 75,689 (38,924-124,628) | 1,320.8 (679.2-2,174.8) | 17,446 (8,966-27,308) | 304.4 (156.5-476.5) | 58,243 (29,958-97,320) | 1016.3 (522.8-1,698.2) |
| ≥5 | 169,628 (57,690-396,326) | 351.0 (119.4-820.2) | 44,163 (13,568-88,217) | 91.4 (28.1-182.6) | 125,465 (44,122-308,109) | 259.6 (91.3-637.6) |
| All | 245,317 (96,614-520,954) | 453.9 (178.7-963.8) | 61,609 (22,534-115,525) | 114 (41.7-213.7) | 183,708 (74,080-405,429) | 339.9 (137.1-750.1) |
| **Age** | **Parainfluenza virus type 2-associated ILI** | | | | | |
|  | **Total** | | **Medically attended** | | **Non-medically attended** | |
|  | **Number (95% CI)** | **Rate^a^ (95% CI)** | **Number (95% CI)** | **Rate^a^ (95% CI)** | **Number (95% CI)** | **Rate^a^ (95% CI)** |
| <1 | 5,322 (537-13,363) | 456.1 (45.6-1145.3) | 1,217 (127-2,947) | 104.3 (10.3-252.5) | 4,105 (411-10,416) | 351.8 (35.7-892.7) |
| 1-4 | 7,597 (761-19,481) | 166.5 (16.9-426.9) | 1,756 (173-4,287) | 38.5 (3.7-93.9) | 5,841 (582-15,194) | 128.0 (12.5-332.9) |
| 5-24 | 58,756 (12,490-120,867) | 295.5 (62.8-607.9) | 14,000 (2,949-26,978) | 70.4 (14.8-135.7) | 44,756 (9,541-93,889) | 225.1 (48.0-472.2) |
| 25-44 | 10,662 (1,074-41,777) | 63.5 (6.7-248.7) | 3,214 (323-9,558) | 19.1 (2.1-56.9) | 7,448 (750-32,219) | 44.3.0 (4.5-191.8) |
| 45-64 | 8,335 (847-39,896) | 96.7 (9.3-462.9) | 2,379 (235-8,059) | 27.6 (3.0-93.5) | 5,956 (593-31,837) | 69.1 (7.0-369.4) |
| ≥65 | 27,373 (2,758-78,343) | 906.2 (8.9-2593.5) | 6,498 (651-17,738) | 215.1 (2.3-587.2) | 20,875 (2,093-60,605) | 691.1 (68.9-2006.3) |
| <5 | 12,919 (1,301-32,843) | 225.4 (22.8-573.1) | 2,973 (291-7,233) | 51.9 (5.3-126.2) | 9,946 (951-25,610) | 173.6 (17.6-446.9) |
| ≥5 | 105,126 (12,490-280,883) | 217.6 (25.8-581.3) | 26,091 (2,949-62,333) | 54.0 (6.1-129) | 79,035 (9,541-218,550) | 163.6 (19.7-452.3) |
| All | 118,045 (12,490-313,725) | 218.4 (23.1-580.4) | 29,064 (2,949-69,566) | 53.8 (5.5-128.7) | 88,981 (9,541-244,159) | 164.6 (17.7-451.7) |
| **Age** | **Parainfluenza virus type 3-associated ILI** | | | | | |
|  | **Total** | | **Medically attended** | | **Non-medically attended** | |
|  | **Number (95% CI)** | **Rate^a^ (95% CI)** | **Number (95% CI)** | **Rate^a^ (95% CI)** | **Number (95% CI)** | **Rate^a^ (95% CI)** |
| <1 | 43,391 (25,477-67,223) | 3,718.9 (2,183.5-5761.4) | 9,912 (5,841-14,539) | 849.5 (500.6-1246.0) | 33,479 (19,636-52,684) | 2,869.3 (1,682.9-4,515.3) |
| 1-4 | 45,173 (24,314-74,823) | 989.8 (532.8-1,639.5) | 10,443 (5,598-16,476) | 228.8 (122.6-361.0) | 34,730 (18,716-58,347) | 761.0 (410.1-1,278.5) |
| 5-24 | 100,424 (38,230-182,412) | 505.1 (192.3-917.5) | 23,926 (8,883-40,772) | 120.3 (44.7-205.1) | 76,498 (29,347-141,640) | 384.8 (147.6-712.4) |
| 25-44 | 74,409 (41,300-170,010) | 442.9 (245.8-1,012.0) | 22,429 (9,497-37,577) | 133.5 (56.5-223.7) | 51,980 (31,803-132,433) | 309.4 (189.3-788.3) |
| 45-64 | 11,847 (1,183-49,782) | 137.5 (13.9-577.6) | 3,382 (341-10,520) | 39.2 (4.1-122.1) | 8,465 (849-39,262) | 98.2 (9.7-455.6) |
| ≥65 | 9,729 (974-43,524) | 322.1 (32.5-1,440.9) | 2,310 (237-10,376) | 76.5 (7.8-343.5) | 7,419 (731-33,148) | 245.6 (2.5-1,097.4) |
| <5 | 88,564 (49,790-142,046) | 1,545.4 (868.8-2,478.7) | 20,355 (11,438-31,015) | 355.2 (199.6-541.2) | 68,209 (38,352-111,031) | 1,190.3 (669.2-1,937.5) |
| ≥5 | 196,409 (79,530-445,727) | 406.5 (164.6-922.4) | 52,047 (18,380-99,244) | 107.7 (38.0-205.4) | 144,362 (61,150-346,483) | 298.8 (126.5-717.0) |
| All | 284,973 (129,319-587,772) | 527.2 (239.3-1,087.4) | 72,402 (29,818-130,258) | 133.9 (55.2-241.0) | 212,571 (99,501-457,514) | 393.3 (184.1-846.4) |

Abbreviations: CI: confidence intervals.

^a^ Rates reported per 100,000 population.

**Table S9: National estimates of mean annual number and rate of parainfluenza virus types 1-3-associated severe respiratory illness (SRI) in South Africa, 2013-2015**

| **Age** | **Parainfluenza virus type 1-associated SRI** | | | | | |
| --- | --- | --- | --- | --- | --- | --- |
|  | **Total** | | **Medically attended** | | **Non-medically attended** | |
|  | **Number (95% CI)** | **Rate^a^ (95% CI)** | **Number (95% CI)** | **Rate^a^ (95% CI)** | **Number (95% CI)** | **Rate^a^ (95% CI)** |
| <1 | 3,462 (1,336-5,677) | 296.7 (114.5-486.6) | 1,599 (616-2,536) | 137.0 (52.8-217.3) | 1,863 (720-3,141) | 159.7 (61.7-269.2) |
| 1-4 | 3,256 (1,300-5,690) | 71.3 (28.5-124.7) | 1,506 (628-2,520) | 33.0 (13.7-55.2) | 1,750 (672-3,170) | 38.3 (14.7-69.5) |
| 5-24 | 1,979 (24-4,986) | 10.0 (0.1-25.1) | 919 (11-2,190) | 4.6 (0.1-11) | 1,060 (13-2,796) | 5.3 (0.1-14.1) |
| 25-44 | 3,672 (1,292-6,749) | 21.9 (7.7-40.2) | 1,715 (658-3,091) | 10.2 (3.9-18.4) | 1,957 (634-3,658) | 11.6 (3.8-21.8) |
| 45-64 | 308 (42-1,285) | 3.6 (0.5-14.9) | 143 (21-558) | 1.7 (0.2-6.5) | 165 (21-727) | 1.9 (0.2-8.4) |
| ≥65 | 495 (27-2,838) | 16.4 (0.9-94.0) | 228 (15-1,300) | 7.5 (0.5-43) | 267 (12-1,538) | 8.8 (0.4-50.9) |
| <5 | 6,718 (2,636-11,366) | 117.2 (46-198.3) | 3,105 (1,244-5,055) | 54.2 (21.7-88.2) | 3,613 (1,392-6,311) | 63.0 (24.3-110.1) |
| ≥5 | 6,454 (1,384-15,857) | 13.4 (2.9-32.8) | 3,005 (705-7,138) | 6.2 (1.5-14.8) | 3,449 (679-8,719) | 7.1 (1.4-18) |
| All | 13,172 (4,020-27,222) | 24.4 (7.4-50.4) | 6,110 (1,949-12,193) | 11.3 (3.6-22.6) | 7,062 (2,071-15,029) | 13.1 (3.8-27.8) |
| **Age** | **Parainfluenza virus type 2-associated SRI** | | | | | |
|  | **Total** | | **Medically attended** | | **Non-medically attended** | |
|  | **Number (95% CI)** | **Rate^a^ (95% CI)** | **Number (95% CI)** | **Rate^a^ (95% CI)** | **Number (95% CI)** | **Rate^a^ (95% CI)** |
| <1 | 356 (31-1,046) | 30.5 (2.7-89.6) | 165 (15-466) | 14.1 (1.3-39.9) | 191 (16-580) | 16.4 (1.4-49.7) |
| 1-4 | 865 (19-2,106) | 19.0 (0.4-46.1) | 399 (9-962) | 8.7 (0.2-21.1) | 466 (10-1,144) | 10.2 (0.2-25.1) |
| 5-24 | 809 (23-2,878) | 4.1 (0.1-14.5) | 376 (11-1,362) | 1.9 (0.1-6.8) | 433 (12-1,516) | 2.2 (0.1-7.6) |
| 25-44 | 1,167 (61-2,924) | 6.9 (0.4-17.4) | 545 (30-1,349) | 3.2 (0.2-8.0) | 622 (31-1,575) | 3.7 (0.2-9.4) |
| 45-64 | 1,775 (49-4,096) | 20.6 (0.6-47.5) | 823 (22-1,881) | 9.5 (0.3-21.8) | 952 (27-2,215) | 11.0 (0.3-25.7) |
| ≥65 | 1,069 (28-4,135) | 35.4 (0.9-136.9) | 491 (15-1,936) | 16.3 (0.5-64.1) | 578 (13-2,199) | 19.1 (0.4-72.8) |
| <5 | 1,221 (50-3,152) | 21.3 (0.9-55.0) | 564 (24-1,428) | 9.8 (0.4-24.9) | 657 (26-1,724) | 11.5 (0.5-30.1) |
| ≥5 | 4,820 (161-1,4031) | 10.0 (0.3-29.0) | 2,235 (78-6,527) | 4.6 (0.2-13.5) | 2,585 (83-7,504) | 5.3 (0.2-15.5) |
| All | 6,041 (211-1,7182) | 11.2 (0.4-31.8) | 2,799 (102-7,954) | 5.2 (0.2-14.7) | 3,242 (109-9,228) | 6.0 (0.2-17.1) |
| **Age** | **Parainfluenza virus type 3-associated SRI** | | | | | |
|  | **Total** | | **Medically attended** | | **Non-medically attended** | |
|  | **Number (95% CI)** | **Rate^a^ (95% CI)** | **Number (95% CI)** | **Rate^a^ (95% CI)** | **Number (95% CI)** | **Rate^a^ (95% CI)** |
| <1 | 14,051 (9,665-19,140) | 1,204.3 (828.3-1,640.4) | 6,489 (4,685-8,355) | 556.1 (401.5-716.0) | 7,562 (4,980-10,785) | 648.1 (426.8-924.3) |
| 1-4 | 6,756 (3,909-10,451) | 148.0 (85.7-229.0) | 3,125 (1,877-4,576) | 68.5 (41.1-100.3) | 3,631 (2,032-5,875) | 79.6 (44.5-128.7) |
| 5-24 | 738 (23-2,548) | 3.7 (0.1-12.8) | 342 (11-1,106) | 1.7 (0.1-5.6) | 396 (12-1,442) | 2.0 (0.1-7.3) |
| 25-44 | 1,672 (67-3,817) | 10.0 (0.4-22.7) | 781 (30-1,758) | 4.6 (0.2-10.5) | 891 (37-2,059) | 5.3 (0.2-12.3) |
| 45-64 | 1,711 (47-3,936) | 19.9 (0.5-45.7) | 792 (21-1,778) | 9.2 (0.2-20.6) | 919 (26-2,158) | 10.7 (0.3-25) |
| ≥65 | 2,467 (30-6,841) | 81.7 (1.0-226.5) | 1,135 (15-3,161) | 37.6 (0.5-104.6) | 1,332 (15-3,680) | 44.1 (0.5-121.8) |
| <5 | 20,807 (13,572-29,591) | 363.1 (236.8-516.4) | 9,614 (6,561-12,931) | 167.8 (114.5-225.6) | 1,1193 (7,011-16,660) | 195.3 (122.3-290.7) |
| ≥5 | 6,588 (166-17,141) | 13.6 (0.3-35.5) | 3,050 (77-7,802) | 6.3 (0.2-16.1) | 3,538 (89-9,339) | 7.3 (0.2-19.3) |
| All | 27,395 (13,738-46,732) | 50.7 (25.4-86.5) | 12,664 (6,638-20,733) | 23.4 (12.3-38.4) | 14,731 (7,100-25,999) | 27.3 (13.1-48.1) |

Abbreviations: CI: confidence intervals.

^a^ Rates reported per 100,000 population

**REFERENCES**

1. Tempia S, Walaza S, Moyes J, et al. [Quantifying How Different Clinical Presentations, Levels of Severity, and Healthcare Attendance Shape the Burden of Influenza-associated Illness: A Modeling Study From South Africa.](https://pubmed.ncbi.nlm.nih.gov/30508065/) Clin Infect Dis. **2019**;69(6):1036-1048. doi: 10.1093/cid/ciy1017. [↑](#endnote-ref-1)
2. Rudan I, Boschi-Pinto C, Biloglav Z, et al. Epidemiology and etiology of childhood pneumonia. Bull World Health Organ. **2008**;86(5):408-16. [↑](#endnote-ref-2)
